# CO_2_ Adsorption and Storage on Ca-Modified Biochar Derived from Coconut Endocarp

**DOI:** 10.1021/acsomega.6c02339

**Published:** 2026-08-25

**Authors:** Emily F. Garcia, Mariana C. Santoro, Daniel F. Cipriano, José G. A. Rodrigues, Jair C. C. Freitas

**Affiliations:** † Laboratory of Carbon and Ceramic Materials (LMC), Department of Physics, Federal University of Espírito Santo (UFES), Vitória 29075-910, ES, Brazil; ‡ Soil and Water Systems Department, University of Idaho, Moscow, Idaho 83844, United States

## Abstract

Carbon capture and storage are technologies capable of reducing atmospheric CO_2_ levels, contributing to mitigating the problems caused by global warming and climate change. Biochar has been widely investigated as an adsorbent for CO_2_ capture due to its low cost and wide availability. However, research on CO_2_ capture followed by storage using biochar is still limited. Therefore, this work aimed to investigate the capture and storage of CO_2_ in physically activated and Ca-modified biochars derived from coconut endocarp. The adsorption and carbonation performances were evaluated at different temperatures and moisture conditions. The maximum adsorption capacity was observed at 30 °C and among the Ca-modified samples, the biochar with the lowest Ca content (3.5 wt %) exhibited the highest specific surface area (577 m^2^/g) and adsorption capacity (46.1 mg/g). The adsorption kinetics were evaluated using three kinetic models, with the Avrami model showing the best fit to the experimental data. Thermal treatment at 510 °C led to the formation of CaCO_3_ particles deposited throughout the porous carbon structure; the sample with the highest Ca content (23.9 wt %) and the lowest specific surface area (242 m^2^/g) showed the highest carbonation capacity (104.6 mg/g). Carbonation of the Ca-modified biochars was also affected by moisture, being significantly faster in humid environments as compared to dry ones at room temperature. The results of this study demonstrated that Ca-modified biochars are environmentally friendly hybrid adsorbents capable of adsorbing and storing CO_2_ in the form of carbonates, potentially serving as a sustainable alternative to help achieving the goals set by the Paris Agreement.

## Graphical abstract

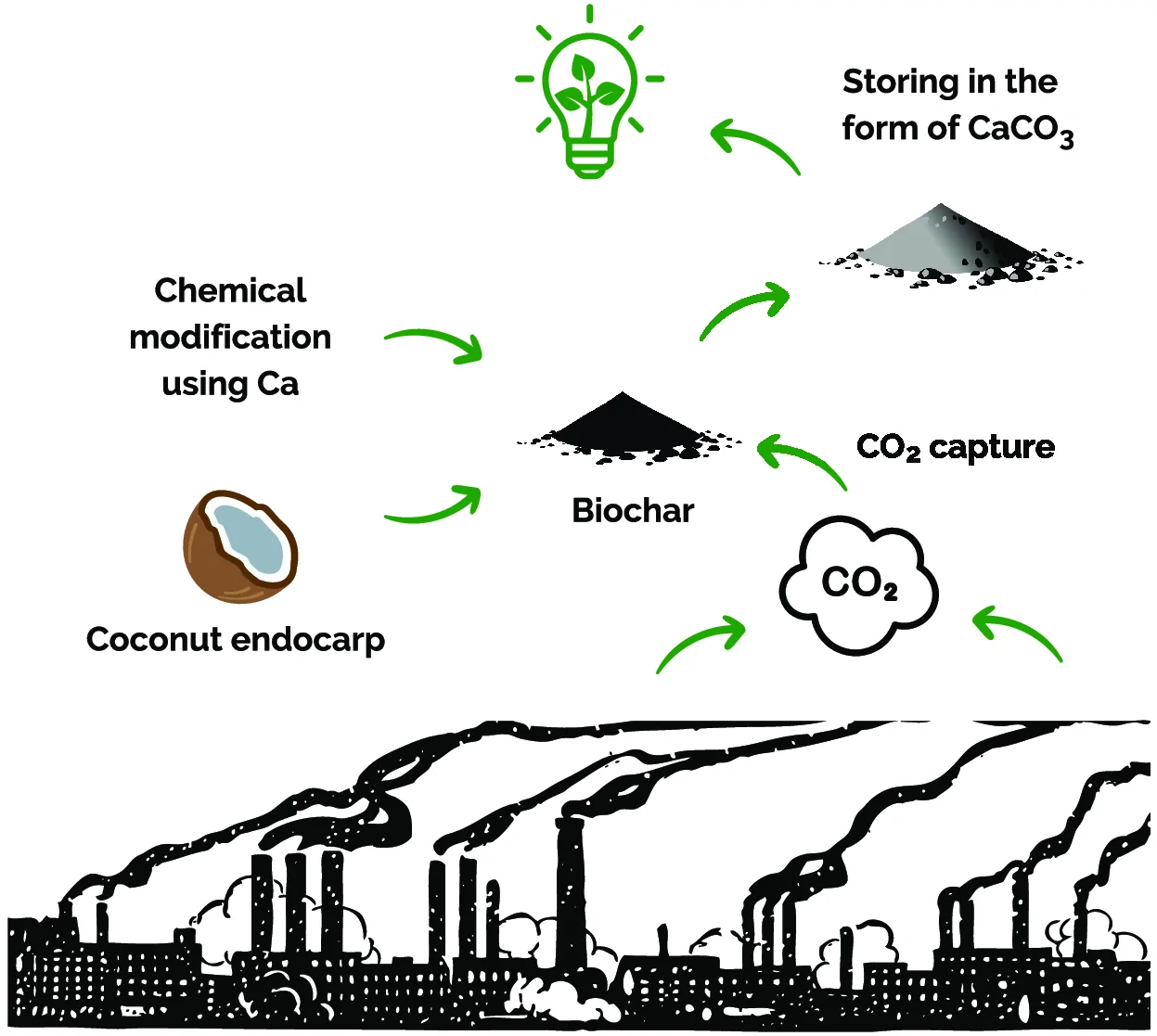

## 1 Introduction

Carbon dioxide (CO_2_) is the primary greenhouse gas (GHG) emitted as a consequence of human activities, such as the burning of fossil fuels, biomass combustion, and industrial processes.^1^ With the continuous advancement of the global economy and population growth, human activities have continued to increase, leading to a significant rise in GHG emissions, contributing to global warming and climate change.^2–4^ In this context, the Paris Agreement, adopted at the 21st Conference of the Parties (COP 21), proposed actions to reduce CO_2_ emissions, aiming to keep the global average temperature increase below 2 °C above preindustrial levels and to limit the temperature increase to 1.5 °C by 2100.^5^

In order to achieve these targets, various strategies have been explored, including reducing energy consumption, transitioning to low- or zero-carbon energy sources, and adopting carbon capture and storage (CCS) technologies.^6^ Among these strategies, CCS technologies are considered indispensable for reducing CO_2_ levels in the medium- and long-term, particularly when the captured CO_2_ is used as a raw material for generating industrial fuels and chemical products.^6,7^ The CCS process involves, in general, three main stages: separation, transportation, and storage of CO_2_.^7,8^ The separation stage, which represents around two-thirds of the total cost, is the biggest challenge for the application of this technology on a large scale, despite being essential for significantly reducing anthropogenic CO_2_ emissions.^8^

Currently, chemical absorption using amines is the most widely used technique for the capture of CO_2_. However, this process has several disadvantages, such as high energy costs and amine leakage.^9^ As an alternative, adsorption on porous materials has been considered a viable option due to its simplicity, low cost, and good adsorbent regeneration capacity.^10^ Various adsorbents have been investigated for CO_2_ capture, including activated carbon (AC), biochar (BC), zeolites, carbon nanotubes, alkali-metal-based materials, graphene-based materials, silica, and metal–organic frameworks.^8,11,12^ Among these materials, physical adsorbents such as AC and BC are more advantageous than chemical adsorbents because, in repeated cycles, they do not form new chemical bonds between CO_2_ and the adsorbent, resulting in lower energy requirements for adsorbent regeneration.^13,14^

AC and BC are carbon-based adsorbents that, although they share similar characteristics, differ mainly in the raw materials used in their production. AC can be produced from fossil residues and biomass, while BC is produced exclusively from biomass.^15–18^ AC is obtained through the carbonization of raw materials followed by physical or chemical activation. On the other hand, BC is obtained through the pyrolysis of biomass, which can also be followed by physical or chemical activation in order to improve the porosity of the material.^16,19–21^ Comparing these two materials, the energy consumption for AC production can be more than 15 times higher than that required for BC production depending on the precursor material and the temperature of production.This difference is due to the carbonization and pyrolysis processes involved in the production of each material.^22^

BCs have garnered significant interest due to their low cost, wide availability, and high thermal stability.^20,22^ The CO_2_ adsorption capacity of BCs depends on their physicochemical and textural properties, such as specific surface area (SSA), pore volume, pore size distribution, and surface basicity/acidity.^15,23^ A study conducted by Cao et al.^24^ evaluated the CO_2_ adsorption capacity of BCs produced from seven types of biomass: rapeseed straw, soybean straw, corn stalk, wheat straw, walnut shell, walnut wood, and pine wood. The results demonstrated that BCs derived from pine wood and walnut wood achieved better pore structures and higher SSA than BCs obtained from straws. Consequently, these materials exhibited higher CO_2_ adsorption capacities, with values of 41.23 and 45.85 mg/g, respectively. In another study, Creamer et al.^25^ evaluated the CO_2_ adsorption capacity of BCs derived from sugarcane bagasse and walnut wood. The BC derived from sugarcane bagasse, produced at 600 °C, exhibited a lower SSA than the BC obtained from walnut wood. However, due to the presence of nitrogen groups on its surface, the BC from sugarcane bagasse showed a higher CO_2_ adsorption capacity at 25 °C, reaching 73.55 mg/g.^25^

In CCS technology, CO_2_ storage includes various methods, such as geological storage, oceanic storage, and mineral carbonation.^26,27^ Mineral carbonation is a CO_2_ storage method that occurs through a gas–solid reaction between CO_2_ and alkaline sorbents, including oxides (such as MgO and CaO), hydroxides (such as Mg­(OH)_2_ and Ca­(OH)_2_), and metastable powdered silicates (such as CaSiO_3_ and Li_2_SiO_3_).^26,28^ During this process, a protective carbonate layer forms around the reacting particles, which may reduce the adsorbent’s SSA.^28^

The modification of BCs with metal compounds, such as potassium (K), calcium (Ca), and magnesium (Mg) compounds, can promote the formation of functional groups on the surface of the BC, potentially contributing to an increase in CO_2_ adsorption capacity or the storage of CO_2_ in the form of carbonates.^28–31^ Bai et al.^29^ investigated the CO_2_ adsorption capacity of BC derived from coconut shell impregnated with KOH, varying the BC/KOH ratio between 0.3 and 1.0. They observed that the sample activated at 750 °C with a BC/KOH ratio of 0.5 achieved a high CO_2_ adsorption capacity, with values of 5.92 mmol/g at 0 °C and 4.15 mmol/g at 25 °C. The study conducted by Ghemei and Behroozi^31^ evaluated the CO_2_ capture capacity of adsorbents based on Mg­(OH)_2_ and Ca­(OH)_2_ under different conditions of temperature, initial pressure, and adsorbent weight. The results showed that Ca­(OH)_2_ exhibited higher efficiency in capturing CO_2_ in the form of carbonate at 35 °C, with an adsorption capacity of 75.19 mg/g, using 1 g of adsorbent and an initial pressure of 4 bar.^31^

Considering the aspects discussed above, the development of an efficient adsorbent for capturing and storing CO_2_ is essential to sustainable development. Faced with this challenge, this work aimed to study CO_2_ capture in BCs derived from the coconut endocarp (CE). These BCs were physically activated and chemically modified with the introduction of Ca­(OH)_2_ particles in their structure. The materials were characterized to evaluate their chemical compositions, morphological characteristics, and textural properties. Subsequently, the efficiency of CO_2_ adsorption and carbonation was assessed under different temperatures and moisture conditions, indicating the potential of Ca-modified BCs for CO_2_ capture and conversion. The obtained results demonstrate, then, that it is possible to develop environmentally friendly hybrid porous adsorbents capable of adsorbing and storing CO_2_ in the form of carbonates.

## 2 Materials and Methods

### 2.1 Materials

#### 2.1.1 Preparation of BC

For the preparation of BC, the carbonization and activation of ground CE were carried out using steam under a continuous flow of argon in a horizontal tubular furnace, using a homemade activation apparatus.^32^ In the carbonization process, the CE was heat-treated at 800 °C at a heating rate of 5 °C/min. After reaching 800 °C, steam was introduced into the furnace for 1 h, with the injection of liquid water controlled by a peristaltic pump. At the end of the physical activation process, the steam flow was turned off and the furnace was allowed to cool naturally down to room temperature under a continuous flow of argon.^33^

#### 2.1.2 Modification of BC with Ca­(OH)_2_

The chemical modification of the CE-derived BC samples was performed by coprecipitation.^33^ Aqueous solutions of CaCl_2_ were mixed with 2 g aliquots of BC, and an aqueous NaOH solution was dropped gradually into the mixture of CaCl_2_ and BC, in order to raise the pH of the reaction medium to ∼12 and allow the precipitation of Ca­(OH)_2_. The mixture was kept under magnetic stirring at 300 rpm for 1 h at room temperature. The concentrations of the used CaCl_2_ and NaOH solutions are provided in Table S1 (Supporting Information). The mixtures were vacuum-filtered, and the obtained Ca-modified BCs were dried at 105 °C in an oven for 16 h. The Ca-modified BC samples with different Ca contents analyzed in this work were named BC_CE/Ca_1_, BC_CE/Ca_2_, BC_CE/Ca_3_, BC_CE/Ca_4_, as detailed in Table S1. For benchmarking, a sample of Ca­(OH)_2_ was also synthesized, following the same procedure above but without the presence of BC in the mixture; this sample was named Ca­(OH)_2__synt.

### 2.2 Characterization

#### 2.2.1 X-ray Diffraction (XRD)

XRD experiments were conducted using a Shimadzu XRD-6000 powder diffractometer, with Cu–Kα radiation (λ = 1.5418 Å) and the following parameters: voltage: 40 kV; current: 30 mA; 2θ angle: from 10 to 80°; scanning speed: 2°/min. In order to assess the chemical stability and to evaluate the carbonation extent of the prepared samples, the XRD analyses were conducted under five different conditions: (I) immediately after sample preparation; (II) after thermal decomposition of the BC_CE/Ca_4_ sample at 500 and 800 °C; (III) after CO_2_ adsorption analysis at 50 °C; (IV) after CO_2_ adsorption analysis followed by thermal treatment; (V) after different time intervals (from one to 7 days) during the carbonation tests conducted at room temperature under different moisture conditions for the BC_CE/Ca4 sample (see details in Section 2.6).

#### 2.2.2 Scanning Eléctron Microscopy (SEM) and Energy-Dispersive X-ray Spectroscopy (EDS)

SEM images were obtained for the BC_CE, BC_CE/Ca_1_, BC_CE/Ca_2_, BC_CE/Ca_3_, and BC_CE/Ca_4_ samples, both before and after thermal treatment at 510 °C, using a Jeol JSM-6610LV scanning electron microscope operating at an acceleration voltage of 20 kV. The SEM images were recorded at 1000× magnification using a secondary electron detector, with samples coated with gold. The EDS spectra were recorded using a Bruker XFlash accessory coupled to the SEM instrument.

#### 2.2.3 Textural Analysis

The textural properties of the prepared samples were analyzed through N_2_ adsorption experiments conducted at 77 K using a Micro200C analyzer from Altamira Instruments. Powder samples, with masses around 0.2 g, were purged under vacuum for 3 h at 200 °C and carefully weighed after the analyses. The SSA values were determined using the Brunauer, Emmett, and Teller (BET) method,^34^ with 6 points selected in the *P*/*P*_0_ range from approximately 0.05 to 0.30. The total pore volume (*V*_P_) was determined directly from the amount of N_2_ adsorbed at the maximum relative pressure (0.99).^35^ The average pore width (*w*) was estimated assuming cylindrical pore geometry and using the relation *w* = 4 × *V*_P_/SSA.^36^ For two representative samples, complete N_2_ adsorption/desorption isotherms were recorded in the relative pressure (*P*/*P*_0_) range from 0.009 to 0.99, using a Micromeritics ASAP 2020 instrument. The pore size distributions were calculated from the adsorption isotherms using the nonlocal density functional theory (NLDFT) approach.^36^

#### 2.2.4 Content of Inorganic Phases

The contents of inorganic phases in the Ca-modified BCs were determined by thermogravimetry (TG), using a Shimadzu DTG-60H instrument. The experiments were conducted in triplicate, with ∼5 mg of samples placed in a platinum crucible and heated at a rate of 10 °C/min up to 900 °C under a continuous flow of N_2_:O_2_, with a flow rate of 50 mL/min for each gas.^37–39^ The content of inorganic phases was determined from the TG curves on a dry basis, considering the residual weight measured at 900 °C (i.e., after combustion of the carbonaceous matrix).

For the analysis of the inorganic phases, two aliquots of 40 mg of the BC_CE/Ca_4_ sample were subjected to thermal decomposition in a muffle furnace, reaching temperatures of 500 and 800 °C, called BC_CE/Ca_4__500 and BC_CE/Ca_4__800. After reaching these temperatures, the samples were cooled in a desiccator to room temperature and analyzed by XRD, as described in Section 2.2.1.

### 2.3 CO_2_ Adsorption

CO_2_ adsorption experiments were conducted at atmospheric pressure using a Shimadzu DTG-60H thermogravimetric analyzer. Approximately 10 mg of the samples were placed in alumina crucibles. Initially, the samples were heated at a rate of 10 °C/min up to 120 °C under a N_2_ flow (50 mL/min), maintaining this temperature constant for 30 min. Then, the temperature was reduced to 30, 50, 80, 100, or 120 °C at a cooling rate of 5 °C/min. Preliminary tests were conducted to determine the stabilization times for each temperature selected for the adsorption study, which were the following: 160 min for 30 °C, 70 min for 50 °C, 60 min for 80 °C, and 40 min for 100 °C. At 120 °C, no additional stabilization time was required. After these intervals, the CO_2_ flow was introduced into the system, maintaining a gas mixture of N_2_:CO_2_ with a flow rate of 50 mL/min for each gas for 10 min until the CO_2_ adsorption stabilized. At the end of the process, the CO_2_ flow was interrupted, and the furnace was allowed to cool naturally to room temperature. The uncertainty in the CO_2_ adsorption capacity was estimated by performing the TG analyses in triplicate for some representative samples, resulting in an uncertainty of ca. 1.0 mg/g. For comparison purposes, the Ca­(OH)_2__synt sample was also subjected to the CO_2_ adsorption experiment at 50 °C.^25,40^

The reversibility of the CO_2_ adsorption process was also evaluated for selected samples using the same TG apparatus. For these experiments, approximately 10 mg of the samples were placed in an alumina crucible and heated at a rate of 10 °C/min to 120 °C under a flow of N_2_ (50 mL/min), maintaining this temperature for 15 min. The temperature was then reduced to 50 °C at a cooling rate of 5 °C/min and maintained for 70 min. After this interval, the CO_2_ adsorption/desorption cycle began. The CO_2_ flow was introduced into the system, maintaining a mixture of N_2_:CO_2_ gases (50 mL/min for each gas) for 10 min, until CO_2_ adsorption stabilized. Then, the CO_2_ flow was interrupted, and the system remained under N_2_ flow (50 mL/min) for 20 min. This procedure was repeated for two more consecutive cycles. At the end of the process, the instrument was cooled naturally to room temperature.

### 2.4 CO_2_ Adsorption Kinetics

The kinetics of CO_2_ adsorption at different temperatures were evaluated by nonlinear fitting using the OriginPro software. Several kinetic models were initially tested; the best results (discussed here) were obtained using the pseudo-first order, pseudo-second order, and Avrami kinetic models.^41,42^ The pseudo-first order model describes a reversible interaction between the adsorbate and the adsorbent and is suitable for predicting the physical adsorption of CO_2_ on solid adsorbents.^42^ The general equation of the pseudo-first order model is represented by^42^

1 $$q_{t}=q_{e}\left(1-\exp\left(-K_{1}t\right)\right)$$

In this expression, *q_t_* (mg g^–1^) is the amount of adsorbed species at time *t* (min), *q*_e_ (mg g^–1^) is the amount of adsorbed species at equilibrium, and *K*_1_ (min^–1^) is the kinetic constant of the pseudo-first-order model.^43^

The pseudo-second-order model describes the adsorption process as occurring through strong bonds between CO_2_ molecules and the adsorbent surface, making it suitable for predicting the chemical adsorption of CO_2_.^41,42^ The general equation of the pseudo-second-order model is represented by^42^

2 $$q_{t}=\frac{K_{2}q_{e}^{2}t}{1+K_{2}q_{e}t}$$

In this expression, *q_t_* (mg g^–1^) is the amount of adsorbed species at time *t* (min), *q*_e_ (mg g^–1^) is the amount of adsorbed species at equilibrium, and *K*_2_ (min^–1^) is the kinetic constant of the pseudo-second-order model.^43^

The Avrami kinetic model is frequently used in studies of phase transitions and crystal growth processes in materials.^44,45^ More recently, this model has also been used to describe other kinetic processes, including CO_2_ adsorption on modified porous adsorbents.^46–49^ The general equation of the Avrami model is represented by^30^

3 $$q_{t}=q_{e}\left(1-\exp\left(-{\left(K_{A}t\right)}^{nA}\right)\right)$$

In this expression, *q_t_* (mg g^–1^) is the amount of adsorbed species at time *t* (min), *q*_e_ (mg g^–1^) is the amount of adsorbed species at equilibrium, *K*_A_ (min^–1^) is the kinetic constant of the Avrami model, and *n*_A_ is the Avrami exponent.^30^ For the fitting tests, the kinetic data were time-corrected to start from the instant when CO_2_ adsorption on the BCs was already in progress. The values of the amount of adsorbed species at time *t* (*q_t_* (mg/g)) were normalized from 0 to 1 relative to the maximum adsorption values (*q*_max_), and new curves of *q_t_*/*q*_max_ vs time were plotted. The nonlinear fittings using the pseudo-first order, pseudo-second order, and Avrami kinetic models were performed on these curves.^42,50^

### 2.5 CO_2_ Adsorption Followed by Carbonation

The experiments of CO_2_ adsorption followed by carbonation were conducted using a Shimadzu DTG-60H thermogravimetric analyzer. Approximately 10 mg of the samples were placed in a platinum crucible and heated at a rate of 10 °C/min up to 120 °C under N_2_ flow (50 mL/min), with this temperature kept constant for 30 min. Then, the temperature was reduced to 50 °C at a cooling rate of 5 °C/min and kept constant for 70 min. After this interval, the CO_2_ flow was introduced into the system, maintaining a gas mixture of N_2_:CO_2_ with a flow rate of 50 mL/min for each gas for 30 min until the stabilization of the sample mass (which indicated the end of CO_2_ adsorption). The temperature was then increased at a heating rate of 10 °C/min up to 510 °C, remaining constant for 10 min, which was verified to be sufficient for the completion of the carbonation process. At the end of the process, the CO_2_ flow was interrupted, and the furnace was allowed to cool naturally to room temperature.^25,28,40^

### 2.6 Carbonation Experiments under Different Moisture Conditions

To evaluate the influence of the presence of adsorbed water on the carbonation process of the Ca-modified BCs, carbonation experiments were conducted for the BC_CE/Ca_4_ sample under different moisture conditions.^51,52^ For these tests, aliquots of this sample (∼40 mg) were first dried in an oven at 105 °C for 16 h and subsequently placed in two separate desiccators. In the first, the sample was exposed to a low-humidity environment using silica gel and keeping a continuous air flow. In the second desiccator, the sample was exposed to a high-humidity environment by placing water filled flasks close to the sample and keeping again a constant air flow. The structural changes occurring in these samples (as a consequence of the carbonation process) were followed by recording XRD patterns of the samples after selected time intervals, corresponding to the condition V described in Section 2.2.1. The moisture content of the sample kept in the high-humidity chamber for 1 week was checked by TG, using the same apparatus and procedures described Section 2.2.4.

## 3 Results and Discussion

### 3.1 Structural and Chemical Characterization of the Ca-Modified BC Samples

The X-ray diffractograms of the as-prepared samples (condition I) are shown in Figure 1. The results reveal the presence of Ca­(OH)_2_ and CaCO_3_ (in different relative amounts) in the samples Ca­(OH)_2__synt, BC_CE/Ca_1_, BC_CE/Ca_2_, BC_CE/Ca_3_, and BC_CE/Ca_4_. The presence of Ca­(OH)_2_ confirms that the coprecipitation process was successful, while the presence of CaCO_3_ indicates that the precipitated Ca­(OH)_2_ can slowly carbonate at room temperature when exposed to CO_2_ present in the ambient atmosphere.^53^ It is interesting to note that the formation of CaCO_3_ is particularly perceptible in the case of the BC_EC/Ca_1_ sample (which is the Ca-modified BC with the largest carbon content and, consequently, the highest SSA, as discussed below); this is attributed to the presence of adsorbed water in the porous carbon matrix, as the presence of water in know to facilitate the conversion of Ca­(OH)_2_ into CaCO_3_.^54^ In the Ca­(OH)_2__synt sample, two intense NaCl peaks were also observed around 31.7 and 45.5°, indicating the formation of this compound as a consequence of the reaction between CaCl_2_ and NaOH. In the BC_CE sample, the broad peaks around 26 and 44° were identified, associated with the turbostratic structure of activated carbon.^55^ In the Ca-modified BC samples, a reduction in the relative intensity of these broad peaks is observed as the concentration of CaCl_2_ and NaOH used for chemical modification increases (Table S1).

**Figure 1:**
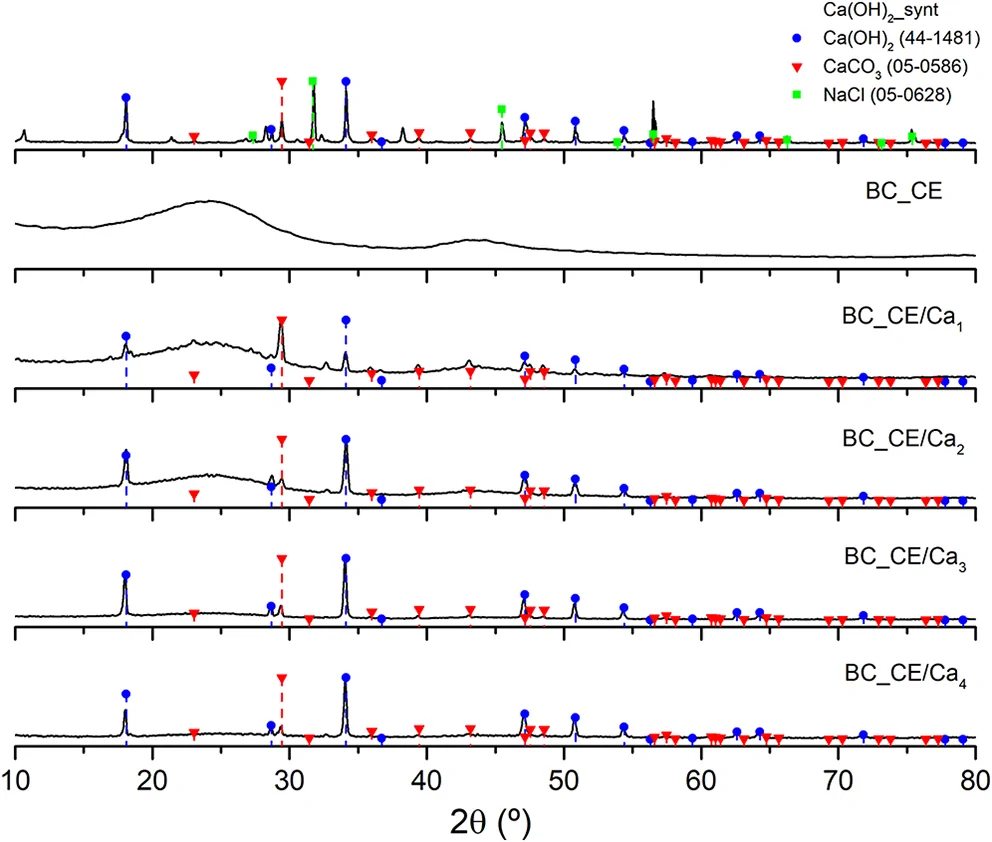
X-ray diffractograms obtained for the as-pared samples Ca­(OH)_2__synt, BC_CE, BC_CE/Ca_1_, BC_CE/Ca_2_, BC_CE/Ca_3_, and BC_CE/Ca_4_.

The surface morphology and chemical properties of the BCs were evaluated using SEM and EDS techniques, as shown in Figures 2, 3, and S1–S6. The BC_CE sample (Figures 2a, S1a, and S2a) exhibits characteristics commonly found in activated BCs, featuring a rough and porous surface with interconnected walls and channels.^32,56,57^ The elemental maps of this sample (Figure S3) reveal that it is primarily composed of carbon, with oxygen-containing particles distributed across the material’s surface.^33^

**Figure 2:**
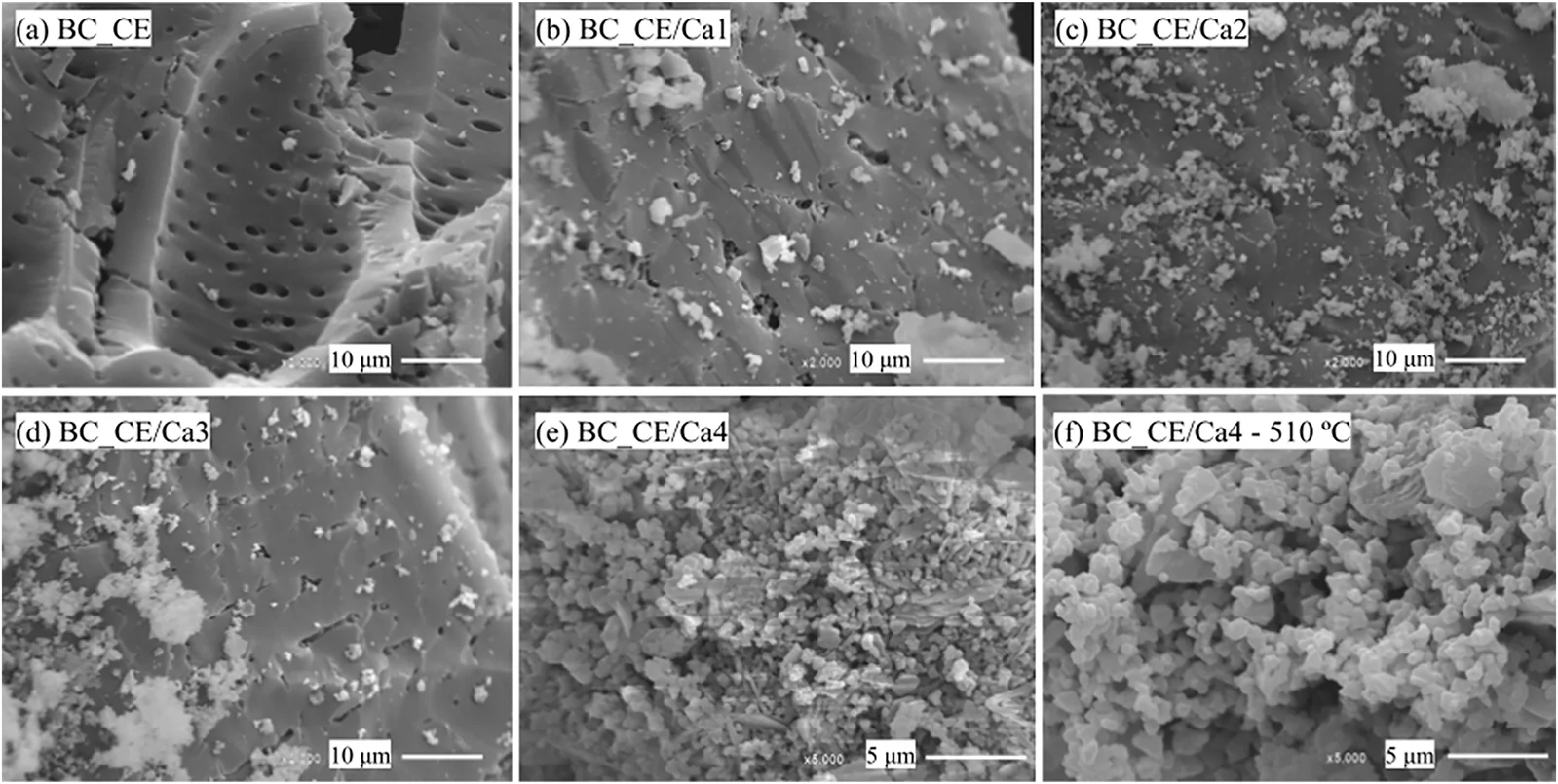
SEM images obtained with different magnifications (2.000 or 5.000×) for the samples: (a) BC_CE, (b) BC_CE/Ca_1_, (c) BC_CE/Ca_2_, (d) BC_CE/Ca_3_, (e) BC_CE/Ca_4_, and (f) BC_CE/Ca_4_ after thermal treatment at 510 °C.

**Figure 3:**
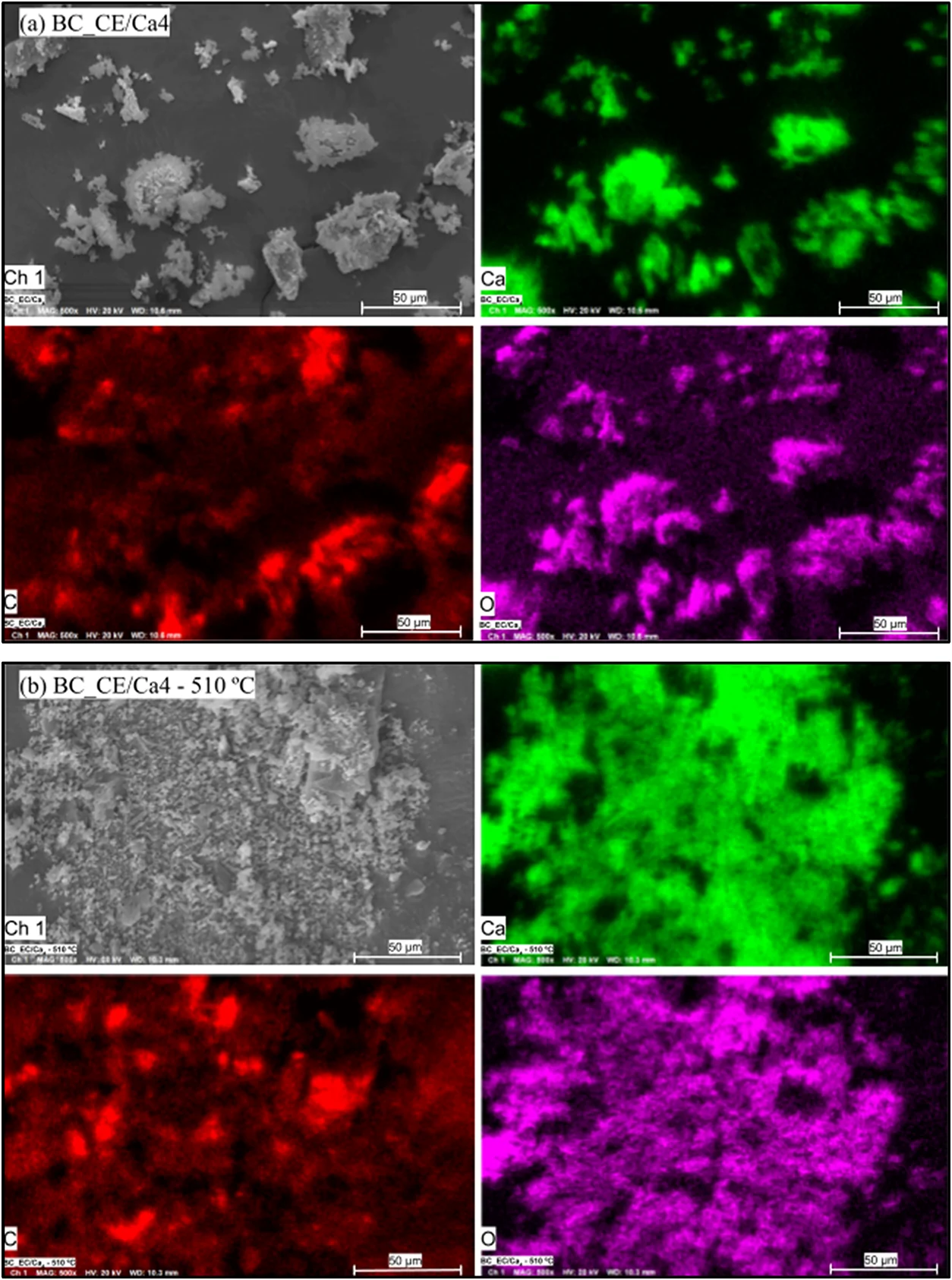
SEM images and the corresponding EDS elemental maps of the BC_CE/Ca_4_ sample before (a) and after (b) thermal treatment at 510 °C (BC_CE/Ca_4_ - 510 °C), corresponding to the elements C, O, and Ca.

The Ca-modified BC samples (Figures 2, S1, and S2) showed similar morphological characteristics, including a porous surface with increased roughness and agglomeration of irregularly shaped, and uniformly distributed particles. The elemental maps obtained for the Ca-modified BCs (Figures 3 and S4–S6) indicated that these samples are primarily composed of carbon with O- and Ca-rich particles distributed across the material’s surface, mostly associated with Ca­(OH)_2_.^58,59^ These results are consistent with the XRD analysis (Figure 1), which identified Ca­(OH)_2_ as the main crystalline phase in the modified BCs. The elemental maps of the BC_CE/Ca_4_ sample after the thermal treatment at 510 °C (Figure 3b) showed a dominant presence of O and Ca, associated with CaCO_3_.^59,60^ As observed in the XRD analysis (Figure 1), after the thermal treatment, Ca­(OH)_2_ underwent carbonation, forming CaCO_3_, as confirmed by the EDS analysis. The comparison between the SEM images exhibited in Figure 2e,2f suggests a thickening of the observed spheroidal particles following the carbonation process, which is attributed to the formation of a CaCO_3_ layer at the surface of the Ca­(OH)_2_ particles that were present before the thermal treatment.^28^

Figure 4 shows TG curves recorded for the BCs. In these curves, the corresponding residual mass values were normalized at 200 °C (i.e., after moisture release) and are thus reported on a dry basis. The BC_CE sample exhibits a significant mass loss around 350–550 °C, associated with the combustion of the carbonaceous matrix,^61^ while the Ca-modified BC samples show two significant mass losses, the first one in the range 350 and 450 °C and the second between 510 and 690 °C. The mass losses observed in the Ca-modified BC samples may be associated with the formation of CaCO_3_ and CaO, as well as the combustion of the carbonaceous matrix.^61,62^

**Figure 4:**
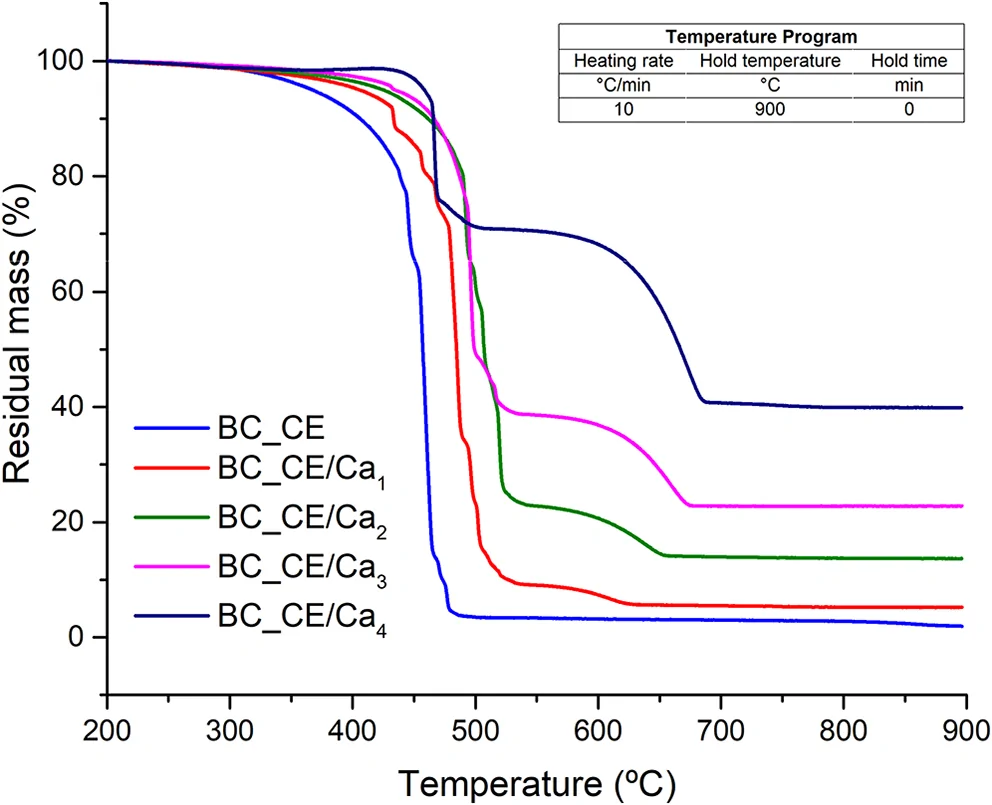
TG curves recorded for the samples BC_CE, BC_CE/Ca_1_, BC_CE/Ca_2_, BC_CE/Ca_3_, and BC_CE/Ca_4_, following the indicated temperature program under a continuous flow of N_2_:O_2_ (50 mL/min for each gas).

To investigate the origin of the first and second mass losses of the Ca-modified samples represented in Figure 4, XRD analyses of the BC_CE/Ca_4_ sample were performed after the thermal decomposition processes at 500 and 800 °C in air in a muffle furnace, as shown in Figure 5.

**Figure 5:**
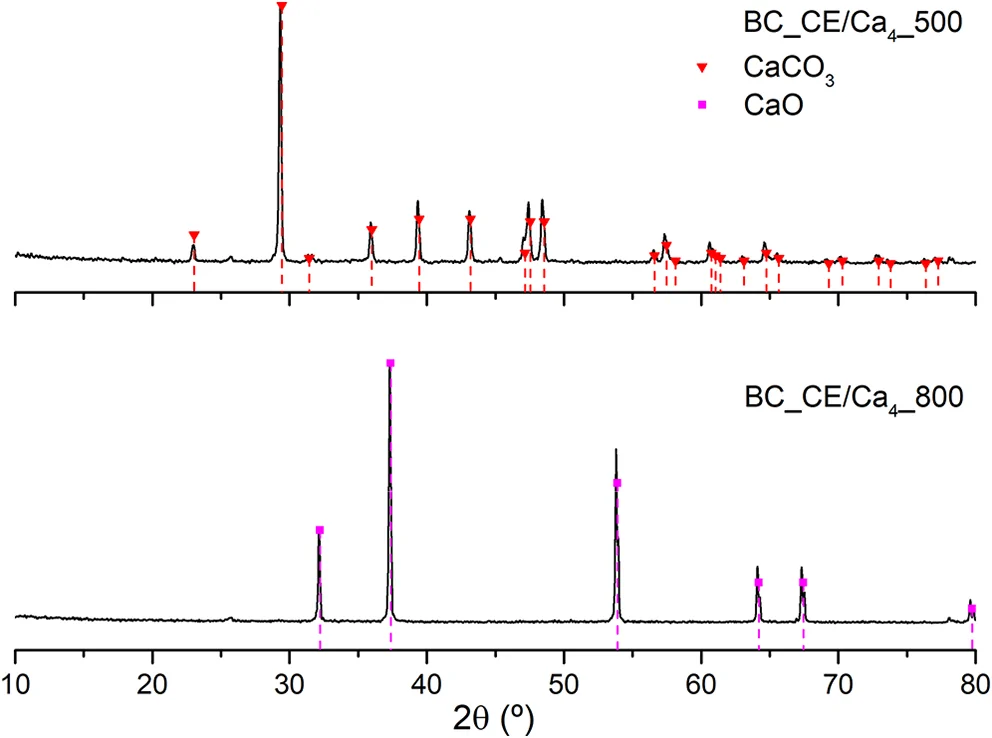
X-ray diffractograms obtained for the BC_CE/Ca_4_ sample after thermal treatments in air at the temperatures of 500 °C (BC_CE/Ca_4__500) and 800 °C (BC_CE/Ca_4__800).

The X-ray diffractogram of the BC_CE/Ca_4__500 sample shows the presence of CaCO_3_, which is due to the carbonation process of Ca­(OH)_2_ present on the surface of the BC, represented by eq 4.^31^ The X-ray diffractogram of the BC_CE/Ca_4__800 sample shows the presence of CaO, formed by the calcination process of CaCO_3_, represented by eq 5.^31^

4 $$\mathrm{Ca}{\left(\mathrm{OH}\right)}_{2}+{\mathrm{CO}}_{2}\to {\mathrm{CaCO}}_{3}+H_{2}O$$

5 $${\mathrm{CaCO}}_{3}\to \mathrm{CaO}+{\mathrm{CO}}_{2}$$

It can then be concluded that, in the thermal decomposition curves of the Ca-modified BCs (Figure 4), the first mass loss is associated with the combustion of the carbonaceous matrix (i.e., activated carbon), which generates CO_2_ and leads concomitantly to the formation of CaCO_3_ (eq 4).^63^ Following this process, the second loss observed in the TG curves shown in Figure 4 indicates the decomposition of CaCO_3_ and the formation of CaO (eq 5).

On the basis of these results, the contents of Ca­(OH)_2_ and Ca in the modified BC samples were estimated from the mass of CO_2_ released during the decomposition of CaCO_3_. First, the difference between the mass after the first loss and the final residual mass was calculated. From this difference, the number of moles of CO_2_ released in the process was calculated, and consequently, the number of moles of Ca­(OH)_2_ was determined using the stoichiometric relationship given in eq 5. From the number of moles of Ca­(OH)_2_, the mass and relative content of Ca­(OH)_2_ and Ca present in the BCs were calculated, considering the total mass of these samples at the beginning of thermal decomposition to be 100%.

The values of Ca­(OH)_2_ and Ca contents of the BC samples, along with the parameters derived from the textural analysis (SSA, *V*_P_, and *w*), are presented in Table 1. As expected, the contents of Ca­(OH)_2_ and Ca precipitated on the surface of the BCs increased gradually with the amounts of CaCl_2_ and NaOH used during the chemical modification reactions (Table S1). For the samples BC_CE/Ca_1_, BC_CE/Ca_2_, and BC_CE/Ca_3_, the concentration of CaCl_2_ increased gradually while the concentration of NaOH remained constant. In the preparation of the BC_CE/Ca_4_ sample, the same concentration of CaCl_2_ as in the case of the BC_CE/Ca_3_ sample was used, with only the concentration of NaOH varying, which contributed to the increased availability of hydroxide ions (OH^–^) and favored the formation and precipitation of Ca­(OH)_2_, as seen in the XRD results (Figure 1).

**Table 1:** Parameters Derived from Textural Analysis (SSA, *V*_P_, and *w*) and the Contents of Ca­(OH)_2_ and Ca Calculated from the TG Curves Obtained for the BC Samples

sample	SSA (m^2^/g)	*V*_P_ (cm^3^/g)	*w* (Å)	Ca(OH)_2_ content (%)	Ca content (%)
BC_CE	634	0.35	22.0		
BC_CE/Ca_1_	577	0.33	22.8	6.3 ± 1.1	3.5 ± 0.6
BC_CE/Ca_2_	494	0.27	22.2	18.1 ± 4.4	10.2 ± 2.4
BC_CE/Ca_3_	342	0.20	23.3	26.0 ± 2.9	14.3 ± 1.6
BC_CE/Ca_4_	242	0.14	22.4	43.8 ± 2.5	23.9 ± 1.4

It is worth noting here that, although the BET method is not the best choice for analyzing the adsorption of N_2_ by mixed microporous/mesoporous adsorbents such as the biochar samples described in this work,^32,33^ this simple method was adopted here just for comparative purposes, in order to evaluate how the Ca modification affected the porosity of the Ca-modified biochar samples. The results given in Table 1 show that as expected, the activated carbon (BC_CE sample) exhibits the highest SSA and *V*_p_ values. In the Ca-modified BC samples, a reduction in the SSA and V_P_ values is observed (with a negligible change in the average pore width) compared to BC_CE, due to the formation and deposition of the Ca­(OH)_2_ crystals throughout the porous carbon matrix. This decrease occurs progressively as the Ca­(OH)_2_ and Ca contents increase, as illustrated in Figure S7. Since Ca­(OH)_2_ has an SSA of less than 6 m^2^/g, its contribution to the total surface area of the Ca-modified BCs is minimal, thus resulting in reduced porosity for all Ca-modified samples;^33,63^ Figure S8a presents the N_2_ adsorption/desorption isotherms of the BC_CE and BC_CE/Ca_4_ samples, which exhibit characteristics of types I and IV isotherms;^64^ the corresponding pore size distributions are shown in Figure S8b. These results evidence a predominantly microporous structure for all analyzed samples (with micropore volume fraction above 90%), with a minor contribution from mesopores. No significant change is noted when comparing the shapes of the isotherms and of the pore size distributions of the BC_CE and the Ca-modified BC samples; only a gradual porosity reduction is observed as a function of the increase in the Ca content, as indicated in Table 1 and in Figure S7.

### 3.2 Evaluation of CO_2_ Adsorption

Figure 6 shows the TG (Figure 6a) and DTA (Figure 6b) curves recorded during CO_2_ adsorption at 50 °C using the BC and Ca­(OH)_2__synt samples. The positive DTA peaks observed in Figure 6b clearly indicate the exothermic nature of CO_2_ adsorption.^65^ This is consistent with a physical adsorption process, where CO_2_ molecules are attracted to the surface of the BCs by van der Waals forces.^66–68^

**Figure 6:**
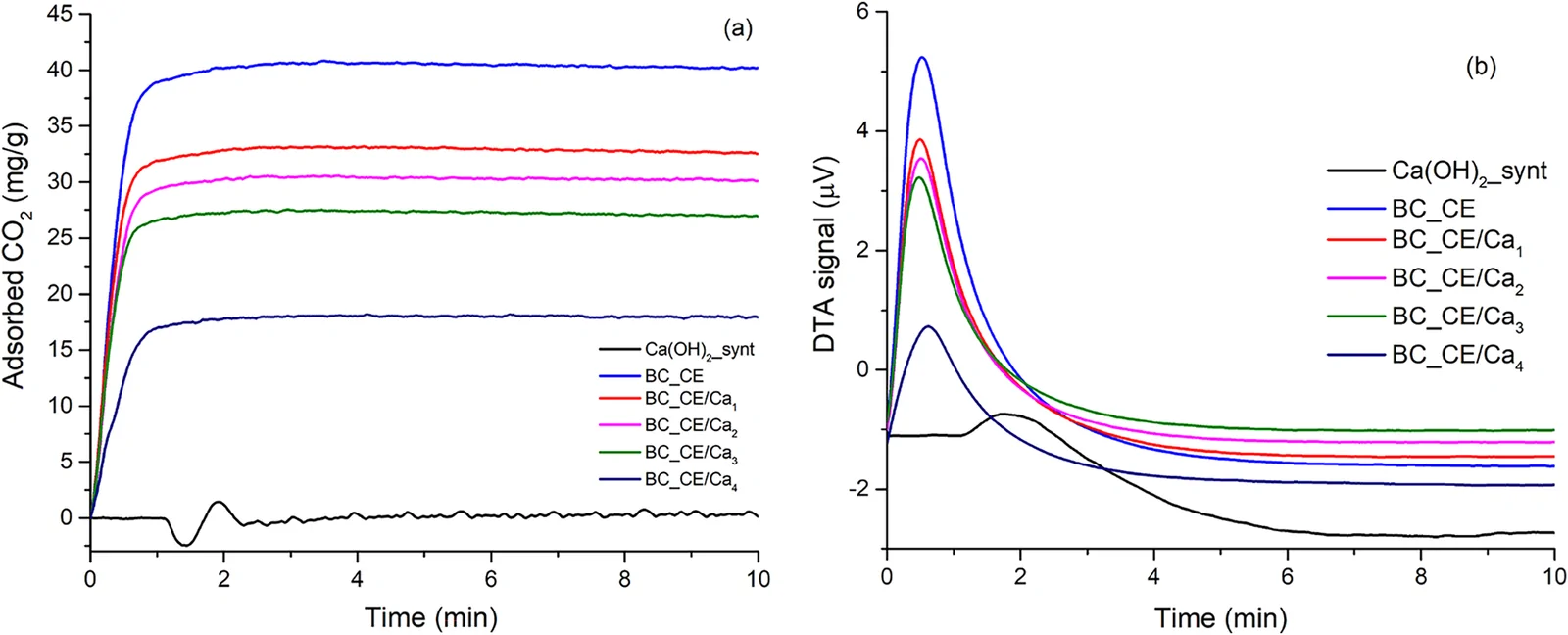
TG (a) and DTA (b) obtained during CO_2_ adsorption at 50 °C for the samples BC_CE, BC_CE/Ca_1_, BC_CE/Ca_2_, BC_CE/Ca_3_, BC_CE/Ca_4_, and Ca­(OH)_2__synt.

The adsorption curves of the BCs exhibit similar behavior with rapid initial adsorption and equilibrium reached within a few minutes. Among the BC samples, BC_CE showed the highest CO_2_ adsorption capacity, while BC_CE/Ca_4_ demonstrated the lowest capacity. This trend is primarily related to the SSA values and, therefore, as discussed above, to the amount of Ca­(OH)_2_ deposited in the porous carbon matrix. As the Ca content increases, the SSA and porosity decrease (Table 1), reducing the adsorption capacities of the BCs. Thus, the higher the Ca content deposited in the porous carbon matrix, the lower is the CO_2_ adsorption capacity. The adsorption curve of the Ca­(OH)_2__synt sample does not show any significant mass increase (with only a slight mass fluctuation observed), indicating that Ca­(OH)_2_ does not have the capacity to physically adsorb CO_2_.

The highest adsorption capacities of the BC samples were obtained at 30 °C, with the *q*_max_ values at this temperature presented in Table S2. To quantitatively assess the relationships between SSA, Ca content, and CO_2_ adsorption capacity, a Pearson correlation analysis was conducted. The results (summarized in Tables S3 and S4) revealed a strong negative correlation between SSA and Ca content (*r* = −0.982), indicating that increasing calcium incorporation significantly reduces the surface area.

In contrast, the CO_2_ adsorption capacity exhibited a strong positive correlation with SSA (r = +0.980), highlighting the key role of porosity on the physical adsorption process. Additionally, a strong negative correlation was observed between Ca content and CO_2_ adsorption capacity (*r* = −0.976). All of these correlations were found to be statistically significant, with *p* < 0.005 (Table S4). These findings demonstrate that the decrease in the CO_2_ adsorption capacity is primarily associated with the porosity reduction, mostly due to the incorporation of nonporous Ca­(OH)_2_ particles into the Ca-modified BC samples.^36,69,70^

Figure 7 presents the TG curves obtained for CO_2_ adsorption at different temperatures (30, 50, 80, 100, and 120 °C) for the BC samples. As in the adsorption experiment conducted at 50 °C (Figure 6a), the BC_CE sample exhibited the highest adsorption capacity at all temperatures, while the BC_CE/Ca_4_ sample showed the lowest capacity, confirming that the adsorption capacity of these samples is mainly influenced by the SSA and the Ca­(OH)_2_ content. In Figure 7, it is also observed that the adsorption capacity of all samples progressively decreases with increasing temperature. This behavior is consistent with the exothermic nature of the CO_2_ adsorption process.^71^

**Figure 7:**
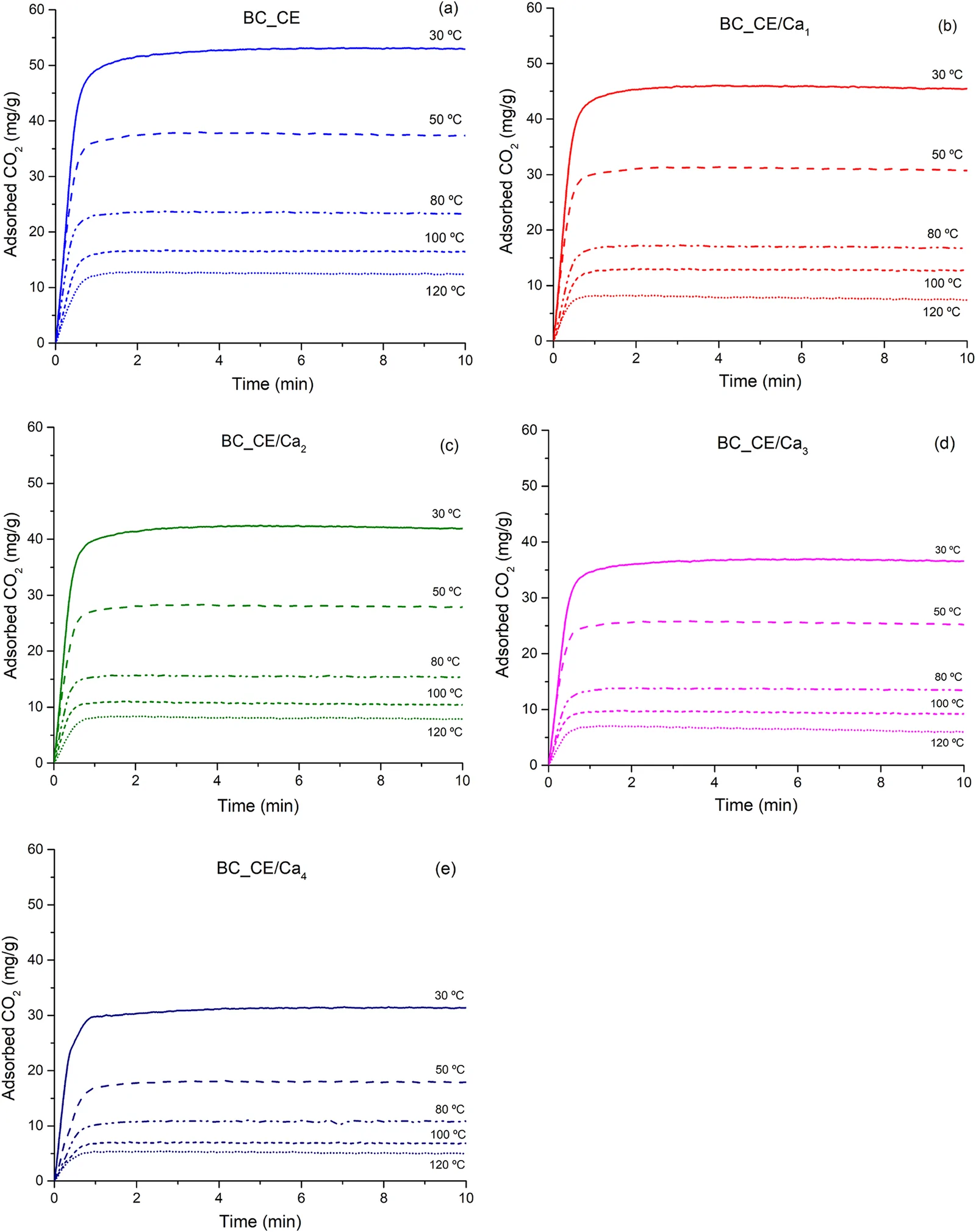
CO_2_ adsorption curves at 30, 50, 80, 100, and 120 °C for the samples (a) BC_CE, (b) BC_CE/Ca_1_, (c) BC_CE/Ca_2_, (d) BC_CE/Ca_3_, and (e) BC_CE/Ca_4_.

It is also worth observing that this physisorption process can be easily reversed and thus the adsorbents described in the present work can be quickly regenerated after CO_2_ adsorption, simply by changing the atmosphere used in the sorption experiments. The TG curves showing three consecutive cycles of CO_2_ adsorption/desorption at 50 °C for two representative samples (BC_CE and BC_CE/Ca_4_) are shown in Figure S9. All of the displayed curves are quite similar, with fast adsorption when the CO_2_ flow is introduced into the system and equilibrium reached within a few minutes, remaining constant. This demonstrates that the adsorption capacity remains stable across consecutive cycles, with values of 40.9, 40.3, and 40.5 mg/g for the first, second, and third cycles, respectively, for BC_CE, and 21.7, 21.4, and 21.1 mg/g for BC_CE/Ca_4_. Desorption is equally fast when the CO_2_ flow is interrupted, leaving only the inert gas (N_2_) flow. This trend confirms that the physical adsorption process is reversible and that the adsorbents are easily regenerated.

From Figure 7, the maximum amount of CO_2_ adsorbed at equilibrium (*q*_max_) was determined for the samples at each analyzed temperature, as shown in Figure 8. This plot shows clearly that for each BC, the *q*_max_ values decrease with increasing temperature. As the adsorption temperature increases, the energy of the CO_2_ molecules also increases, making the adsorbed molecules unstable and thereby reducing the interaction between CO_2_ and the BC surface.^70–72^

**Figure 8:**
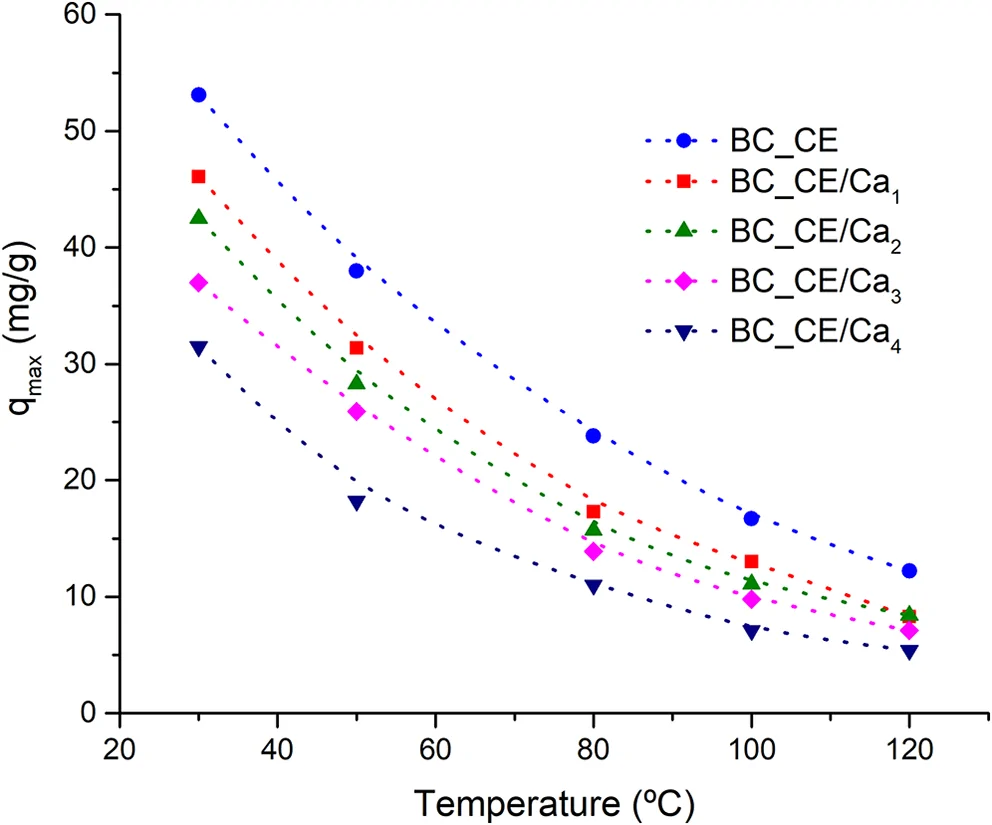
Maximum CO_2_ adsorption capacity (*q*_max_) as a function of temperature for the samples BC_CE, BC_CE/Ca_1_, BC_CE/Ca_2_, BC_CE/Ca_3_, and BC_CE/Ca_4_. The dashed lines are guides to the eyes.

For comparative purposes, Table 2 presents examples of different biochar samples, prepared from varied sources and including, in some cases, chemical modifications, along with their CO_2_ adsorption capacities at atmospheric pressure and specific surface areas. It is clear that there is a high scattering of the adsorption capacities, which are mostly dependent on the biochar porosity but are also affected by other factors, such as the mineral content of the material and the nature of the lignocellulosic precursor. For example, the sample derived from coconut endocarp (BC_CE) described here exhibits lower SSA (634 m^2^/g) and adsorption capacity (53.1 mg/g) compared to the biochar obtained from coconut shell (1177 m^2^/g and 260.54 mg/g).^29^ In general, the SSA significantly influences the CO_2_ adsorption capacity. Another example is that the Ca-modified biochar in this study (BC_CE/Ca_4_) exhibited a reduction in the CO_2_ adsorption capacity, associated with a decrease in its SSA in comparison to the original biochar (from 634 to 242 m^2^/g). In Mg-modified biochar, a reduction in porosity also occurs; however, the incorporation of Mg compensates for the SSA decrease due to the increased basicity of the material, enhancing its affinity for CO_2_ and, consequently, its adsorption capacity.^30,50^ In addition, the comparison given in Table 2 shows that the BC samples described in this work exhibit adsorption capacities comparable to those of the other mentioned biochar-based adsorbents, even considering that these capacities were evaluated at atmospheric pressure in the present work. Although the deposition of Ca­(OH)_2_ particles in biochar reduces the physisorption capacity, this chemical modification enables the hybrid character of the material, integrating adsorption and carbonation. As explained before, there is a significant reduction in the adsorption capacity of the BC_CE/Ca_4_ sample in comparison with the BC_CE sample due to the reduction in the porosity caused by the presence of the Ca­(OH)_2_ particles in the former case; on the other hand, the Ca modification grants an important carbonation ability to this hybrid adsorbent (as detailed below).

**Table 2:** Comparison of the CO_2_ Adsorption Performance of Various Biochars at Atmospheric Pressure

biomass precursor	adsorption temperature (°C)	SSA (m^2^/g)	adsorption capacity (mg/g)	remarks about preparation conditions	ref
sugarcane bagasse	25	388	73.55	pyrolysis at 600 °C	^25^
wheat straw	25	20	34.40	pyrolysis at 500 °C	^74^
rambutan peel	30	597 (pristine) 505 (modified)	68.74 (pristine) 78.80 (modified)	pyrolysis at 900 °C, impregnation with Mg salts	^30^
walnut shell	25	397 (pristine) 292 (modified)	72.60 (pristine) 82.00 (modified)	pyrolysis at 900 °C, impregnation with Mg salts	^69^
seeds of the date fruit	20	798	141.14	pyrolysis at 800 °C, physical activation with CO_2_ at 900 °C	^75^
coconut shell	0	1177	260.54	KOH activation at 750 °C, mass ratio KOH/CS of 0.5:1	^29^
coconut endocarp	30	634 (pristine)^a^ 242 (modified)^b^	53.1 (pristine)^a^ 31.5 (modified)^b^	pyrolysis and physical activation with steam at 800 °C, modification with Ca	this work

a BC_CE sample.

b BC_CE/Ca_4_ sample.

The CO_2_ adsorption curves shown in Figure 7 were analyzed using different kinetic models, as described in Section 2.4. The fitting curves obtained from the use of these models at different temperatures are presented in Figures S10–S14, with the corresponding fitting parameters given in Tables S5–S9. It is observed that the *R* ^2^ values of the Avrami model are, in general, higher than those obtained for the pseudo-first-order and pseudo-second-order models, which agrees with the visual inspection of the matching between the experimental data and the fitting curves shown in Figures S10–S14. Therefore, the Avrami kinetic model is considered the best model to fit the experimental curves shown in Figure 7, and it is thus reasonable to use the parameters derived from this model for a comparative analysis of the kinetic behavior of the Ca-modified BC samples when used as CO_2_ adsorbents at different temperatures.

The better fitting obtained using the Avrami kinetic model suggests that the CO_2_ adsorption process exhibits a more complex kinetic behavior, characterized by multiple steps involving mass transfer (surface/intraparticle diffusion) and surface interactions.^49^

In general, the Avrami exponent (*n*_A_) obtained for each sample increased with the adsorption temperature and remained close to 1, indicating that the CO_2_ adsorption process occurs uniformly over the entire surface of the BC, which is characteristic of homogeneous adsorption, in which all regions of the surface have an equal probability of being occupied by CO_2_ molecules.^42,73^ These results are consistent with studies reported in the literature, such as those by Vega et al.^49^ and Monteagudo et al.,^73^ who associated *n*_A_ values close to 1 with homogeneous CO_2_ adsorption processes.

Figure S15 shows the curves of *K*_A_ as a function of the adsorption temperature. A general trend of increasing *K*_A_ values with the increase in temperature can be observed, with no clear dependence on the Ca­(OH)_2_ concentration in the adsorbent. All samples showed *K*_A_ values within the same range (between 2 and 6 min^–1^), and these values are consistent with the literature.^30^ However, it is important to note that the relatively large uncertainties in these *K*_A_ values (∼1 min^–1^, as estimated from triplicate experiments conducted for representative samples) preclude a more detailed analysis of the trends shown in Figure S15. The values of the kinetic constants for the CO_2_ adsorption on porous carbon materials can be influenced by factors such as the type of precursor used to prepare the carbon adsorbent, the material impregnated in the porous matrix, and the CO_2_ concentration.^30,69,76^

### 3.3 CO_2_ Adsorption Followed by Carbonation

Figure 9 shows the curves of CO_2_ adsorption at 50 °C followed by thermal treatment up to 510 °C for the BC samples and the Ca­(OH)_2__synt samples. It is observed that the CO_2_ physisorption at 50 °C is similar to the process described in Section 3.2, with the CO_2_ adsorption capacity decreasing as the Ca­(OH)_2_ contents in the Ca-modified BCs increase. With the rise in the temperature under CO_2_ flow, the TG curves exhibited in Figure 9 show that all BC samples first release the previously adsorbed CO_2_ and then, above 350 °C, the weight gain clearly indicates the occurrence of the carbonation reaction. Thus, under these experimental conditions, CO_2_ adsorption/desorption and carbonation appear as separate and consecutive processes.

**Figure 9:**
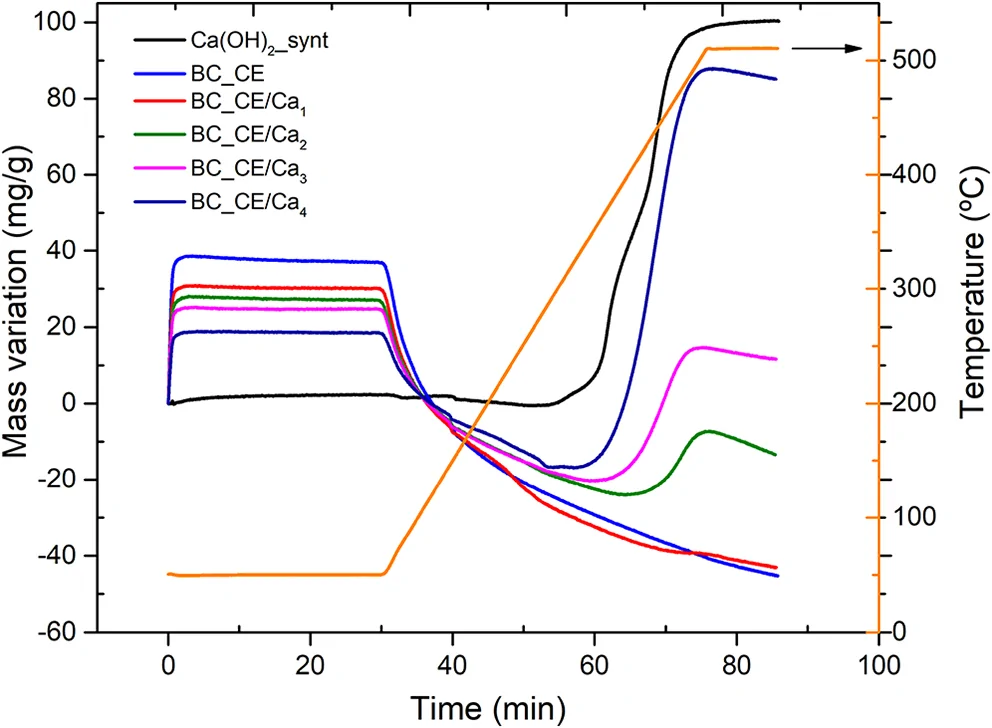
TG curves showing the isothermal CO_2_ adsorption at 50 °C followed by the thermal treatment up to 510 °C under CO_2_ flow (to study the carbonation process), recorded for the samples: BC_CE, CE_EC/Ca_1_, BC_CE/Ca_2_, BC_CE/Ca_3_, and BC_CE/Ca_4_. The orange curve represents the temperature variation during the experiment.

The increase in temperature favored the carbonation process for the Ca-modified samples and for Ca­(OH)_2__synt. Among the Ca-modified BC samples, the carbonation capacity increased as the Ca­(OH)_2_ content increased. It is worth noting that the mass gain due to carbonation was quite limited in the case of the BC_CE/Ca_1_ sample, which is due to the dominant presence of CaCO_3_ in its structure, as shown in the corresponding XRD pattern in Figure 1. As discussed before, this sample is the one with the largest SSA among the Ca-modified BCs, which facilitates the carbonation even at room temperature in a moist environment.^54^

The carbonation of Ca­(OH)_2_ occurs between 350 and 400 °C and, at higher temperatures (500–600 °C), there may be a competition between the dehydroxylation of Ca­(OH)_2_ to CaO and the carbonation of Ca­(OH)_2_ and CaO.^31,77^ The analyses conducted up to 510 °C confirmed that the carbonation process occurred within the favorable temperature range reported in the literature. All of the Ca-modified BC samples exhibited hybrid characteristics, capable of capturing and storing CO_2_ in the form of carbonate. Among the samples exhibiting these hybrid characteristics, the BC_CE/Ca_4_ sample (which is quite porous, in spite of its large Ca content – see Table 1) can be highlighted as the one that exhibits both a fair CO_2_ adsorption capacity at near-ambient temperature and a sizable carbonation capacity at higher temperatures. Although the adsorption capacity of this sample decreased due to its high Ca content and reduced SSA (in comparison with the BC_CE sample), this limitation was compensated for by its considerable carbonation capacity. Furthermore, physical adsorption is a reversible process, whereas carbonation converts CO_2_ into stable carbonates, promoting its permanent storage. Therefore, when the primary objective is long-term CO_2_ capture and storage, the BC_CE/Ca_4_ sample is a promising candidate for use in CCS technologies.

### 3.4 Stability and Changes in XRD Patterns

The stability of the samples was evaluated by XRD analyses conducted under conditions I, III, and IV, i.e., immediately after sample preparation (I), after CO_2_ adsorption at 50 °C (III), and after the carbonation experiments at 510 °C (IV); the results are displayed in Figure S16. The X-ray diffractograms of the Ca­(OH)_2__synt sample (Figure S16a) show that from condition I to condition III, the intensities of the Ca­(OH)_2_ peaks decrease, indicating that the sample slowly carbonates when exposed to CO_2_ present in the ambient atmosphere.

From condition I to condition IV, the Ca­(OH)_2_ peaks disappear, while the CaCO_3_ peaks become more intense, demonstrating that the carbonation process at 510 °C was effective.

In Figure S16b, the XRD patterns of the BC_CE are essentially identical; these patterns are again dominated by the peaks associated with the carbon turbostratic structure, with the presence of only a small peak at 2θ = 26.18° due to SiO_2_ (quartz),^78^ which is known to be present in the EC precursor.^79^

Figure S16 also shows that the XRD patterns of the Ca-modified BC samples are distinct only for the BC_CE/Ca_1_ sample, which, as already mentioned, is the one that already exhibited large carbonation just after preparation; the CaCO_3_ diffraction peaks dominate all of the XRD patterns shown in Figure S16c. For the other Ca-modified samples, the dominant diffraction peaks are due to Ca­(OH)_2_ both immediately after sample preparation and after CO_2_ adsorption, whereas the CaCO_3_ phase is dominant after the carbonation experiments.

These results show then that the CO_2_ adsorption at 50 °C was not accompanied by any significant carbonation, whereas the carbonation process at 510 °C was effective for all of the Ca-modified BCs.

### 3.5 Carbonation Experiments with Humidity Variation

The X-ray diffractograms of the BC_CE/Ca_4_ sample under experimental conditions I (i.e., immediately after sample preparation) and V (after different time intervals during the carbonation tests conducted at room temperature under different moisture conditions) are presented in Figure 10.

**Figure 10:**
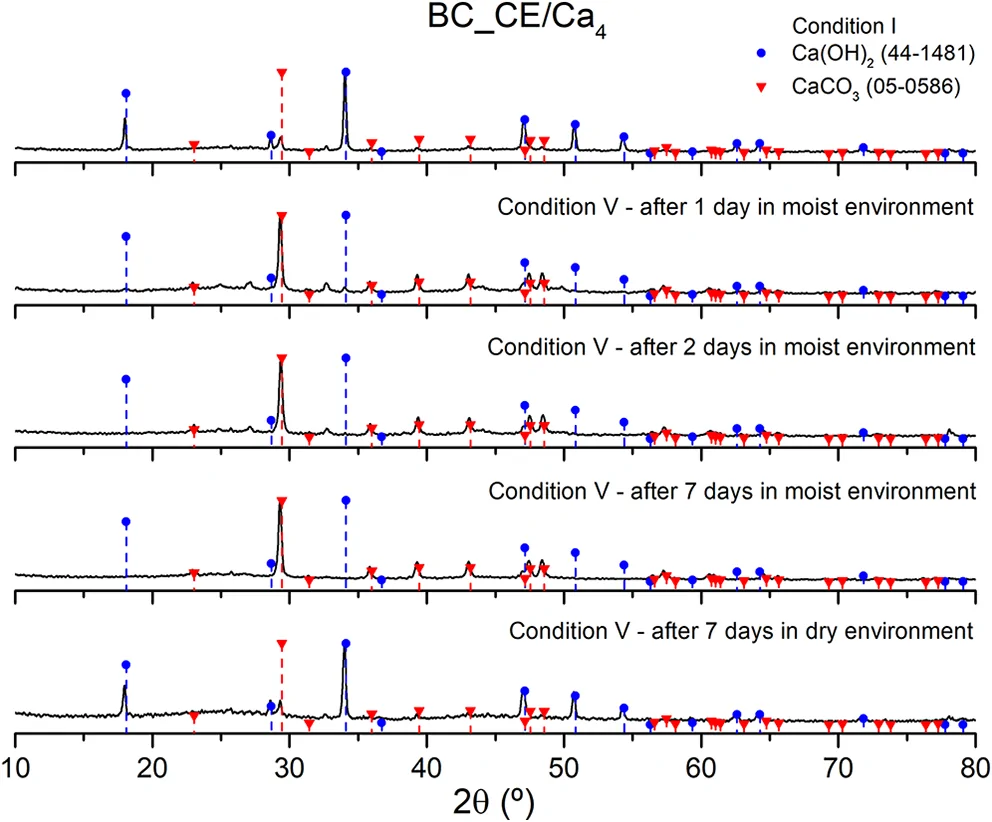
X-ray diffractograms obtained for the BC_CE/Ca_4_ sample under conditions I (immediately after sample preparation) and V (1, 2, and 7 days after carbonation test conducted in dry or moist environments).

The results show that the sample exposed to CO_2_ under dry conditions does not exhibit significant changes over time. On the other hand, for the sample exposed to CO_2_ under humid conditions, the XRD patterns reveal the formation of CaCO_3_ after just 1 day, with no further changes at longer times, indicating that humidity accelerated the carbonation process of Ca­(OH)_2_ present on the sample’s surface. These results are consistent with studies reported in the literature, such as those by Dheilly et al.^54^ and Dostie et al.,^52^ which demonstrate that the presence of water vapor promotes the dissolution of Ca­(OH)_2_, the hydration of CO_2_, and the formation of CaCO_3_.^52,54^ The high porosity and the hydrophilic nature of the activated carbon matrix are thus expected to contribute to facilitating water adsorption. As shown before (Section 3.2), these porous carbon samples are also capable of easily adsorbing CO_2_ at room temperature. The presence of adsorbed water accelerates then the carbonation process by promoting the dissolution of Ca­(OH)_2_ and the hydration of physisorbed CO_2_, leading to the fast carbonation evidenced by the results shown in Figure 10.

As previously observed, at temperatures around room temperature and in environments with limited humidity, CO_2_ adsorption by porous carbons is favored and carbonation of Ca­(OH)_2_ occurs slowly (becoming likelier at temperatures >350 °C). The XRD patterns recorded for condition V (shown in Figure 10) reveal that humidity can compensate for the need for high temperatures to initiate the carbonation process, facilitating the formation of CaCO_3_ at the surface of the Ca­(OH)_2_ particles deposited onto the porous carbon matrix. The sample kept in the high-humidity chamber was analyzed by TG after 1 week (Figure S17), revealing a moisture content of 11.5 wt %. This relatively high moisture content contributed to the acceleration of the carbonation process of Ca­(OH)_2_ present on the BC surface. In the Ca-modified BC samples under moist ambient conditions, physical adsorption of water occurs on the BC porous surface. This adsorbed water facilitates the dissociation of Ca­(OH)_2_ into Ca^2+^ and OH^–^ ions (eq 6).^54^ The OH^–^ ion reacts with CO_2_ dissolved in the adsorbed water (eq 7), forming CO_3_^2–^ and H^+^ ions in a basic medium (pH > 10) (eqs 8 and 9).^52,54,80^

6 $$\mathrm{Ca}{\left(\mathrm{OH}\right)}_{2\left(s\right)}\to {{\mathrm{Ca}}^{2+}}_{\left(\mathrm{aq}\right)}+2{{\mathrm{OH}}^{-}}_{\left(\mathrm{aq}\right)}$$

7 $${\mathrm{CO}}_{2\left(g\right)}+H_{2}O_{\left(l\right)}\to H_{2}{\mathrm{CO}}_{3\left(\mathrm{aq}\right)}$$

8 $$H_{2}{\mathrm{CO}}_{3}\left(\mathrm{aq}\right)\to {H^{+}}_{\left(\mathrm{aq}\right)}+{{{\mathrm{HCO}}_{3}}^{-}}_{\left(\mathrm{aq}\right)}$$

9 $${{\mathrm{HCO}}_{3\left(\mathrm{aq}\right)}}^{-}+{{\mathrm{OH}}^{-}}_{\left(\mathrm{aq}\right)}\to {{{\mathrm{CO}}_{3}}^{2-}}_{\left(\mathrm{aq}\right)}+H_{2}O_{\left(l\right)}$$

Subsequently, the formed CO_3_^2–^ (eq 9 and H)^+^ ions (eq 8) react with the Ca^2+^ and OH^–^ ions produced in eq 3 to form CaCO_3_ and H_2_O (eqs 10 and 11).^52,80^

10 $${{\mathrm{Ca}}^{2+}}_{\left(\mathrm{aq}\right)}+{{{\mathrm{CO}}_{3}}^{2-}}_{\left(\mathrm{aq}\right)}\to {\mathrm{CaCO}}_{3\left(s\right)}$$

11 $${H^{+}}_{\left(\mathrm{aq}\right)}+{{\mathrm{OH}}^{-}}_{\left(\mathrm{aq}\right)}\to H_{2}O_{\left(l\right)}$$

The above-discussed results demonstrate that humidity is an essential factor for the carbonation of Ca-modified BC samples at room temperature, as the relatively large porosity and the hydrophilic character of these samples provide an environment rich in water that facilitates the carbonation process. This suggests that the Ca-containing porous carbon materials described in this work are promising for CCS technologies, as the relatively quick carbonation process at room temperature requires considerably less energy than thermal treatments at high temperatures, where the formation of CaCO_3_ from Ca­(OH)_2_ or CaO is thermodynamically favorable.

Finally, it is worth stressing that mineral carbonation is considered nowadays a promising method of CO_2_ conversion and storage into a stable form (carbonates), whose reversion would require a great deal of thermal energy. Unlike other CCS methods (such as geological and ocean storage), where the retained CO_2_ may leak, mineral carbonation is a permanent and safe way of storing CO_2_ in the form of carbonates, which are stable and environmentally harmless materials that can be used as raw materials in sectors such as construction and agriculture.^26,27,81–84^ Although the cost of the process is considered high in comparison with other CCS strategies, it can be a viable alternative especially when the source of the mineral species to be used for CO_2_ capture comes from industrial residues (such as mining wastes).^81,85,86^ Compared with conventional CO_2_ capture technologies, such as chemical absorption with amines and geological and ocean storage, the materials studied here may offer potential advantages. Chemical absorption with amines, although widely commercialized for its operational simplicity and high CO_2_ capture capacity, presents important drawbacks, including environmental risks and high energy demand for regeneration.^9^ During geological and ocean storage processes, CO_2_ may escape into the atmosphere, potentially causing ecological and geological imbalances.^26^ The materials described here are prepared from low-cost raw materials and involve low energy demand due to the possibility of carbonation under near-ambient conditions, in addition to CO_2_ storage in the form of stable carbonates. However, the practical application of this method still depends on techno-economic assessments, to be addressed in future investigations. The results presented in this work point then to a potentially useful strategy to improve the efficiency of mineral carbonation, as the dispersion of the mineral species into a porous carbon matrix can contribute to accelerating the carbonation process at room temperature.

## 4 Conclusions

This work described an investigation about CO_2_ and conversion capacity of Ca-containing porous biochar samples derived from a biomass precursor, evaluating their performance at variable temperatures and under different humidity conditions. The results obtained showed that temperature, SSA, and Ca­(OH)_2_ content significantly influence the CO_2_ adsorption capacity of the BCs. The material with the smallest Ca content (BC_CE/Ca_1_, with an SSA of 577 m^2^/g and 6.3% Ca­(OH)_2_) exhibited a maximum adsorption capacity of 46.1 mg/g at 30 °C, whereas the sample with the largest Ca content (BC_CE/Ca_4_, with an SSA of 242 m^2^/g and 43.8% of Ca­(OH)_2_) showed a maximum adsorption capacity of 31.5 mg/g. The adsorption capacity decreased with increasing temperature (due to the exothermic nature of the process) and also with the increasing Ca content, due to the reduction in the SSA caused by the deposition of the Ca­(OH)_2_ particles on the porous carbon surface. Among the evaluated kinetic models, the Avrami model showed the best fit to the experimental data of the CO_2_ adsorption process. Thermal treatment at 510 °C showed that carbonation of the Ca-modified BC samples was favored at high temperatures and by the increase in the Ca­(OH)_2_ content, resulting in the formation of CaCO_3_. Carbonation of the Ca-modified BCs was also strongly affected by humidity; the XRD patterns recorded for samples exposed to environments with different humidity levels revealed that the carbonation was considerably faster in humid environments than in dry ones. The large porosity and the hydrophilic nature of the activated carbon matrix contributed to facilitating water adsorption and to speeding up the carbonation process, by promoting the dissolution of Ca­(OH)_2_ and the hydration of physisorbed CO_2_, leading thus to fast carbonation at room temperature. The BC_CE/Ca_4_ sample (which was still reasonably porous, in spite of its large Ca content) exhibited both a fair CO_2_ adsorption capacity at near-ambient temperature and a sizable carbonation capacity, being easily carbonated even at room temperature when exposed to a moist environment.

The results of this work demonstrate that Ca-modified BCs are environmentally friendly hybrid porous materials capable of adsorbing and storing CO_2_ in the form of carbonates, particularly when stored in moist environments. Because of the use of low-cost precursors (coconut endocarp) and the low energy demand required for carbonation under near-ambient conditions, these Ca-containing porous carbon materials represent a potential alternative for use in CCS technologies, offering a promising approach for reducing CO_2_ levels in the atmosphere or in industrial gas streams.

## Supplementary Material



## Notes

Description of the prepared samples (Table S1); specific surface area (SSA), Ca content, and CO_2_ adsorption capacity (*q*_max_) values (Table S2); Pearson correlation matrix and the corresponding *p*-values for SSA, Ca content, and *q*_max_ values (Tables S3 and S4); fitting parameters of the pseudo-first order, pseudo-second order, and Avrami kinetic models for the CO_2_ adsorption process on Ca-modified and unmodified BC samples (Tables S5–S9); SEM micrographs (Figures S1 and S2); EDS elemental maps (Figures S3–S6); comparative plot of SSA, *V*_P_, and Ca contents (Figure S7); N_2_ adsorption/desorption isotherms and pore size distributions (Figure S8); CO_2_ adsorption/desorption cycling tests (Figure S9); fitting curves obtained using the pseudo-first order, pseudo-second order, and Avrami kinetic models for the CO_2_ adsorption process on Ca-modified and unmodified BC samples (Figures S10–S14); Avrami kinetic constants as a function of temperature (Figure S15); X-ray diffractograms obtained under different conditions to investigate the carbonation process (Figure S16); TG curve after the carbonation experiment under humid conditions (Figure S17) (PDF)
