# Supplementary material for: CO2 Adsorption and Storage on Ca-Modified Biochar Derived from Coconut Endocarp
Source: ACS Omega. 2026 Aug 25;11(35):52520–34. doi: 10.1021/acsomega.6c02339 (PMC13564026; doi:10.1021/acsomega.6c02339)

Supporting Information

# $CO_2$ adsorption and storage on Ca-modified biochar derived from coconut endocarp

Emily F. Garcia[1*], Mariana C. Santoro[1,2], Daniel F. Cipriano[1], José G. A. Rodrigues[1], Jair C. C. Freitas[1]

[1]Federal University of Espírito Santo (UFES), Department of Physics, Avenue Fernando Ferrari, 514, 29075-910, Vitória, Brazil.

[2] University of Idaho, Soil and Water Systems Department, 875 Perimeter Dr. MS 2340, 83844, Moscow, USA.

[*] Corresponding author. E-mail address: emily.garcia@edu.ufes.br

**Table S1.** Description of the samples prepared and analyzed in this work.

**Table S2.** Specific surface area (SSA), Ca content, and $CO_2$ adsorption capacity ($q_{max}$) at 30 ºC obtained for the indicated samples.

**Table S3.** Pearson correlation matrix between SSA, Ca content, and $q_{max}$.

**Table S4.** p-values for the Pearson correlations between SSA, Ca content, and $q_{max}$.

**Table S5.** Fitting parameters corresponding to the pseudo-first order, pseudo-second order, and Avrami kinetic models for the $CO_2$ adsorption process on the BC_CE sample at different temperatures.

**Table S6.** Fitting parameters corresponding to the pseudo-first order, pseudo-second order, and Avrami kinetic models for the $CO_2$ adsorption process on the BC_CE/$Ca_1$ sample at different temperatures.

**Table S7.** Fitting parameters corresponding to the pseudo-first order, pseudo-second order, and Avrami kinetic models for the $CO_2$ adsorption process on the BC_CE/$Ca_2$ sample at different temperatures.

**Table S8.** Fitting parameters corresponding to the pseudo-first order, pseudo-second order, and Avrami kinetic models for the $CO_2$ adsorption process on the BC_CE/$Ca_3$ sample at different temperatures.

**Table S9.** Fitting parameters corresponding to the pseudo-first order, pseudo-second order, and Avrami kinetic models for the $CO_2$ adsorption process on the BC_CE/$Ca_4$ sample at different temperatures.

**Figure S1.** Results obtained from the SEM analysis for the samples of (a) BC_CE, (b) BC_CE/$Ca_1$, (c) BC_CE/$Ca_2$, (d) BC_CE/$Ca_3$, (e) BC_CE/$Ca_4$ and (f) BC_CE/$Ca_4$ after heat treatment at 510 ºC, at a magnification of 35 and 40×.

**Figure S2.** Results obtained from the SEM analysis for the samples of (a) BC_CE, (b) BC_CE/$Ca_1$, (c) BC_CE/$Ca_2$, (d) BC_CE/$Ca_3$, (e) BC_CE/$Ca_4$ and (f) BC_CE/$Ca_4$ after heat treatment at 510 ºC, at a magnification of 100 and 250×.

**Figure S3.** EDS elemental maps of the BC_CE sample, corresponding to the C and O elements at 500× magnification.

**Figure S4.** EDS elemental maps of the BC_CE/$Ca_1$ sample, corresponding to the elements C, O and Ca at 500× magnification.

**Figure S5.** EDS elemental maps of the BC_CE/$Ca_2$ sample, corresponding to the elements C, O and Ca at 500× magnification.

**Figure S6.** EDS elemental maps of the BC_CE/$Ca_3$ sample, corresponding to the elements C, O and Ca at 500× magnification.

**Figure S7.** Comparative plot showing the SSA and $V_P$ values together with the Ca contents of the BC samples.

**Figure S8.** (a) $N_2$ adsorption/desorption isotherms and (b) pore size distribution of the BC_CE and BC_CE/$Ca_4$ samples.

**Figure S9.** TG curves recorded during three consecutive cycles of $CO_2$ adsorption at 50 °C using the BC_CE and BC_CE/$Ca_4$ samples.

**Figure S10.** Fitting curves obtained using the pseudo-first order, pseudo-second order, and Avrami kinetic models for the $CO_2$ adsorption process on the BC_CE sample at temperatures of (a) 30, (b) 50, (c) 80, (d) 100, and (e) 120 ºC.

**Figure S11.** Fitting curves obtained using the pseudo-first order, pseudo-second order, and Avrami kinetic models for the $CO_2$ adsorption process on the BC_CE/$Ca_1$ sample at temperatures of (a) 30, (b) 50, (c) 80, (d) 100, and (e) 120 ºC.

**Figure S12.** Fitting curves obtained using the pseudo-first order, pseudo-second order, and Avrami kinetic models for the $CO_2$ adsorption process on the BC_CE/$Ca_2$ sample at temperatures of (a) 30, (b) 50, (c) 80, (d) 100, and (e) 120 ºC.

**Figure S13.** Fitting curves obtained using the pseudo-first order, pseudo-second order, and Avrami kinetic models for the $CO_2$ adsorption process on the BC_CE/$Ca_3$ sample at temperatures of (a) 30, (b) 50, (c) 80, (d) 100, and (e) 120 ºC.

**Figure S14.** Fitting curves obtained using the pseudo-first order, pseudo-second order, and Avrami kinetic models for the $CO_2$ adsorption process on the BC_CE/$Ca_4$ sample at temperatures of (a) 30, (b) 50, (c) 80, (d) 100, and (e) 120 ºC.

**Figure S15.** Avrami kinetic constant as a function of temperature for the samples: (a) BC_CE, (b) BC_CE/$Ca_1$, (c) BC_CE/$Ca_2$, (d) BC_CE/$Ca_3$, and (e) BC_CE/$Ca_4$.

**Figure S16.** X-ray diffractograms obtained under conditions I (immediately after sample preparation), III (after $CO_2$ adsorption at 50 ºC), and IV (after the carbonation experiments at 510 ºC) for the samples: (a) $Ca(OH)_2$_synt, (b) BC_CE, (c) BC_CE/$Ca_1$, (d) BC_CE/$Ca_2$, (e) BC_CE/$Ca_3$ and (f) BC_CE/$Ca_4$.

**Figure S17.** TG curve recorded for the BC_CE/$Ca_4$ sample after the carbonation experiment in a humid environment.

Table S1. Description of the samples prepared and analyzed in this work.

| Samples | Sample mass (g) | $CaCl_2$ solution volume (L) | NaOH solution volume (L) | $CaCl_2$ concentration (g/L) | NaOH concentration (g/L) |
|---|---|---|---|---|---|
| BC_CE | - | - | | - | - |
| BC_CE/$Ca_1$ | 2 | 0.1 | 0.025 | 7.5 | 43.2 |
| BC_CE/$Ca_2$ | 2 | 0.1 | 0.025 | 15.0 | 43.2 |
| BC_CE/$Ca_3$ | 2 | 0.1 | 0.025 | 22.5 | 43.2 |
| BC_CE/$Ca_4$ | 2 | 0.1 | 0.0247 | 22.5 | 64.8 |
| $Ca(OH)_2$_sint | 2 | 0.1 | 0.025 | 22.5 | 43.2 |

Table S2. Specific surface area (SSA), Ca content, and $CO_2$ adsorption capacity ($q_{max}$) at 30 ºC obtained for the indicated samples.

| Samples | SSA ($m^2/g$) | Ca (%) | $q_{max}$ (mg/g) |
|---|---|---|---|
| BC_CE | 634 | - | 53.1 |
| BC_CE/$Ca_1$ | 577 | 3.2 | 46.1 |
| BC_CE/$Ca_2$ | 494 | 10.2 | 42.5 |
| BC_CE/$Ca_3$ | 342 | 14.3 | 37.0 |
| BC_CE/$Ca_4$ | 242 | 23.9 | 31.5 |

Table S3. Pearson correlation matrix between SSA, Ca content, and $q_{max}$.

| Variables | SSA ($m^2/g$) | Ca (%) | $q_{max}$ (mg/g) |
|---|---|---|---|
| SSA ($m^2/g$) | 1 | - | - |
| Ca (%) | -0.982 | 1 | - |
| $q_{max}$ (mg/g) | 0.980 | -0.976 | 1 |

Table S4. p-values for the Pearson correlations between SSA, Ca content and $q_{max}$.

| Variables | SSA ($m^2/g$) | Ca (%) | $q_{max}$ (mg/g) |
|---|---|---|---|
| SSA ($m^2/g$) | - | - | - |
| Ca (%) | 0.00280 | - | - |
| $q_{max}$ (mg/g) | 0.00350 | 0.00445 | - |

Table S5. Fitting parameters corresponding to the pseudo-first order, pseudo-second order, and Avrami kinetic models for the $CO_2$ adsorption process on the BC_CE sample at different temperatures.

| | BC_CE | | | | |
|---|---|---|---|---|---|
| Pseudo-first order | 30 ºC | 50 ºC | 80 ºC | 100 ºC | 120 ºC |
| $q_e^a$ | 0.9831 ± 0.0009 | 0.9871 ± 0.0006 | 0.9850 ± 0.0007 | 0.9822 ± 0.0003 | 1.000 ± 0.002 |
| $K_1$ ($min^{-1}$) | 3.5 ± 1.0 | 3.7 ± 1.0 | 4.7 ± 1.0 | 4.2 ± 1.0 | 2.9 ± 1.0 |
| $R^2$ | 0.966 | 0.994 | 0.996 | 0.997 | 0.993 |
| Pseudo-second order | | | | | |
| $q_e^a$ | 1.0220 ± 0.0008 | 1.037 ± 0.003 | 1.050 ± 0.003 | 1.006 ± 0.002 | 1.139 ± 0.009 |
| $K_2$ ($min^{-1}$) | 6.4 ± 2.2 | 6.5 ± 2.2 | 7.9 ± 2.2 | 8.3 ± 2.2 | 3.4 ± 2.2 |
| $R^2$ | 0.987 | 0.948 | 0.964 | 0.928 | 0.955 |
| Avrami | | | | | |
| $q_e^a$ | 0.9980 ± 0.0004 | 0.986 ± 0.006 | 0.9853 ± 0.0007 | 0.9818 ± 0.0003 | 0.9901 ± 0.0007 |
| $K_A$ ($min^{-1}$) | 2.2 ± 1.0 | 4.0 ± 1.0 | 4.6 ± 1.0 | 4.4 ± 1.0 | 3.6 ± 1.0 |
| $n_A$ | 0.580 ± 0.003 | 1.06 ± 0.1 | 0.98 ± 0.01 | 1.046 ± 0.006 | 1.206 ± 0.007 |
| $R^2$ | 0.993 | 0.995 | 0.996 | 0.997 | 0.999 |

[a] Normalized amount of adsorbed species at equilibrium.

Table S6. Fitting parameters corresponding to the pseudo-first order, pseudo-second order, and Avrami kinetic models for the $CO_2$ adsorption process on the BC_CE/$Ca_1$ sample at different temperatures.

| BC_CE/$Ca_1$ | | | | | |
|---|---|---|---|---|---|
| Pseudo-first order | 30 ºC | 50 ºC | 80 ºC | 100 ºC | 120 ºC |
| $q_e$[a] | 0.9866 ± 0.0008 | 0.9945 ± 0.0007 | 0.9812 ± 0.0005 | 0.9848 ± 0.0008 | 0.994 ± 0.002 |
| $K_1$ ($min^{-1}$) | 4.0 ± 1.0 | 3.9 ± 1.0 | 4.6 ± 1.0 | 4.3 ± 1.0 | 4.5 ± 1.0 |
| $R^2$ | 0.990 | 0.994 | 0.996 | 0.997 | 0.994 |
| Pseudo-second order | | | | | |
| $q_e$[a] | 1.036 ± 0.002 | 1.049 ± 0.003 | 1.030 ± 0.003 | 1.066 ± 0.005 | 1.094 ± 0.008 |
| $K_2$ ($min^{-1}$) | 7.0 ± 2.2 | 6.5 ± 2.2 | 8.4 ± 2.2 | 6.4 ± 2.2 | 6.1 ± 2.2 |
| $R^2$ | 0.971 | 0.946 | 0.946 | 0.958 | 0.946 |
| Avrami | | | | | |
| $q_e$[a] | 0.9889 ± 0.0008 | 0.9909 ± 0.0006 | 0.9814 ± 0.0005 | 0.9829 ± 0.0008 | 0.9888 ± 0.0006 |
| $K_A$ ($min^{-1}$) | 3.5 ± 1.0 | 4.4 ± 1.0 | 4.6 ± 1.0 | 4.7 ± 1.0 | 5.8 ± 1.0 |
| $n_A$ | 0.898 ± 0.010 | 1.102 ± 0.010 | 0.992 ± 0.008 | 1.066 ± 0.009 | 1.188 ± 0.007 |
| $R^2$ | 0.992 | 0.996 | 0.996 | 0.997 | 0.999 |

[a] Normalized amount of adsorbed species at equilibrium.

Table S7. Fitting parameters corresponding to the pseudo-first order, pseudo-second order, and Avrami kinetic models for the $CO_2$ adsorption process on the BC_CE/$Ca_2$ sample at different temperatures.

| BC_EC/$Ca_2$ | | | | | |
|---|---|---|---|---|---|
| Pseudo-first order | 30 ºC | 50 ºC | 80 ºC | 100 ºC | 120 ºC |
| $q_e$[a] | 0.9857 ± 0.0007 | 0.9890 ± 0.0004 | 0.9814 ± 0.0005 | 0.9835 ± 0.0010 | 0.9904 ± 0.0020 |
| $K_1$ ($min^{-1}$) | 3.8 ± 1.0 | 3.8 ± 1.0 | 5.1 ± 1.0 | 4.9 ± 1.0 | 3.6 ± 1.0 |
| $R^2$ | 0.986 | 0.995 | 0.994 | 0.996 | 0.991 |
| Pseudo-secon order | | | | | |
| $q_e$[a] | 1.029 ± 0.002 | 1.025 ± 0.002 | 1.016 ± 0.002 | 1.069 ± 0.006 | 1.091 ± 0.008 |
| $K_2$ ($min^{-1}$) | 6.8 ± 2.2 | 7.4 ± 2.2 | 10.2 ± 2.2 | 7.2 ± 2.2 | 5.0 ± 2.2 |
| $R^2$ | 0.967 | 0.925 | 0.915 | 0.952 | 0.936 |
| Avrami | | | | | |
| $q_e$[a] | 0.9875 ± 0.0007 | 0.9880 ± 0.0003 | 0.9804 ± 0.0004 | 0.9835 ± 0.0001 | 0.9839 ± 0.0008 |
| $K_A$ ($min^{-1}$) | 3.3 ± 1.0 | 4.4 ± 1.0 | 6.0 ± 1.0 | 4.9 ± 1.0 | 4.7 ± 1.0 |
| $n_A$ | 0.90 ± 0.01 | 1.11 ± 0.01 | 1.11 ± 0.01 | 1.00 ± 0.01 | 1.23 ± 0.01 |
| $R^2$ | 0.987 | 0.997 | 0.996 | 0.996 | 0.998 |

[a] Normalized amount of adsorbed species at equilibrium.

Table S8. Fitting parameters corresponding to the pseudo-first order, pseudo-second order, and Avrami kinetic models for the $CO_2$ adsorption process on the BC_CE/$Ca_3$ sample at different temperatures.

| | BC_CE/$Ca_3$ | | | | |
|---|---|---|---|---|---|
| Pseudo-first order | 30 ºC | 50 ºC | 80 ºC | 100 ºC | 120 ºC |
| $q_e$[a] | 0.9822 ± 0.0009 | 0.9895 ± 0.0007 | 0.9852 ± 0.0005 | 0.9839 ± 0.0010 | 0.9870 ± 0.0020 |
| $K_1$ ($min^{-1}$) | 3.8 ± 1.0 | 4.2 ± 1.0 | 4.5 ± 1.0 | 4.2 ± 1.0 | 4.2 ± 1.0 |
| $R^2$ | 0.976 | 0.995 | 0.997 | 0.992 | 0.992 |
| Pseudo-second order | | | | | |
| $q_e$[a] | 1.021 ± 0.001 | 1.050 ± 0.003 | 1.032 ± 0.003 | 1.045 ± 0.005 | 1.093 ± 0.009 |
| $K_2$ ($min^{-1}$) | 7.0 ± 2.2 | 7.0 ± 2.2 | 8.2 ± 2.2 | 7.0 ± 2.2 | 5.6 ± 2.2 |
| $R^2$ | 0.978 | 0.925 | 0.944 | 0.921 | 0.943 |
| Avrami | | | | | |
| $q_e$[a] | 0.9968 ± 0.0004 | 0.9878 ± 0.0007 | 0.9850 ± 0.0005 | 0.9806 ± 0.0005 | 0.9805 ± 0.0010 |
| $K_A$ ($min^{-1}$) | 2.3 ± 1.0 | 4.8 ± 1.0 | 4.6 ± 1.0 | 5.6 ± 1.0 | 5.5 ± 1.0 |
| $n_A$ | 0.58 ± 0.01 | 1.09 ± 0.01 | 1.01 ± 0.01 | 1.22 ± 0.01 | 1.20 ± 0.01 |
| $R^2$ | 0.993 | 0.996 | 0.997 | 0.998 | 0.998 |

[a] Normalized amount of adsorbed species at equilibrium.

Table S9. Fitting parameters corresponding to the pseudo-first order, pseudo-second order, and Avrami kinetic models for the $CO_2$ adsorption process on the BC_CE/$Ca_4$ sample at different temperatures.

| BC_CE/$Ca_4$ | | | | | |
|---|---|---|---|---|---|
| Pseudo-first order | 30 ºC | 50 ºC | 80 ºC | 100 ºC | 120 ºC |
| $q_e^a$ | 0.9828 ± 0.0007 | 0.9854 ± 0.0008 | 0.9760 ± 0.0007 | 0.9799 ± 0.0010 | 0.9761 ± 0.0010 |
| $K_1$ (min$^{-1}$) | 3.7 ± 1.0 | 2.7 ± 1.0 | 2.9 ± 1.0 | 3.0 ± 1.0 | 3.8 ± 1.0 |
| $R^2$ | 0.979 | 0.991 | 0.986 | 0.983 | 0.991 |
| Pseudo-second order | | | | | |
| $q_e^a$ | 1.019 ± 0.001 | 1.045 ± 0.003 | 1.018 ± 0.002 | 1.031 ± 0.004 | 1.037 ± 0.004 |
| $K_2$ (min$^{-1}$) | 6.9 ± 2.2 | 4.5 ± 2.2 | 5.3 ± 2.2 | 5.3 ± 2.2 | 6.4 ± 2.2 |
| $R^2$ | 0.973 | 0.937 | 0.910 | 0.894 | 0.921 |
| Avrami | | | | | |
| $q_e^a$ | 0.9851 ± 0.0007 | 0.9825 ± 0.0007 | 0.9760 ± 0.0010 | 0.9768 ± 0.0006 | 0.9735 ± 0.0008 |
| $K_A$ (min$^{-1}$) | 3.1 ± 1.0 | 3.2 ± 1.0 | 2.9 ± 1.0 | 3.9 ± 1.0 | 4.5 ± 1.0 |
| $n_A$ | 0.85 ± 0.01 | 1.17 ± 0.01 | 1.00 ± 0.01 | 1.30 ± 0.01 | 1.16 ± 0.01 |
| $R^2$ | 0.983 | 0.994 | 1.000 | 0.995 | 0.995 |

[a] Normalized amount of adsorbed species at equilibrium.

Figure S1. Results obtained from the SEM analysis for the samples of (a) BC_CE, (b) BC_CE/$Ca_1$, (c) BC_CE/$Ca_2$, (d) BC_CE/$Ca_3$, (e) BC_CE/$Ca_4$ and (f) BC_CE/$Ca_4$ after heat treatment at 510 ºC, at a magnification of 35 and 40×.

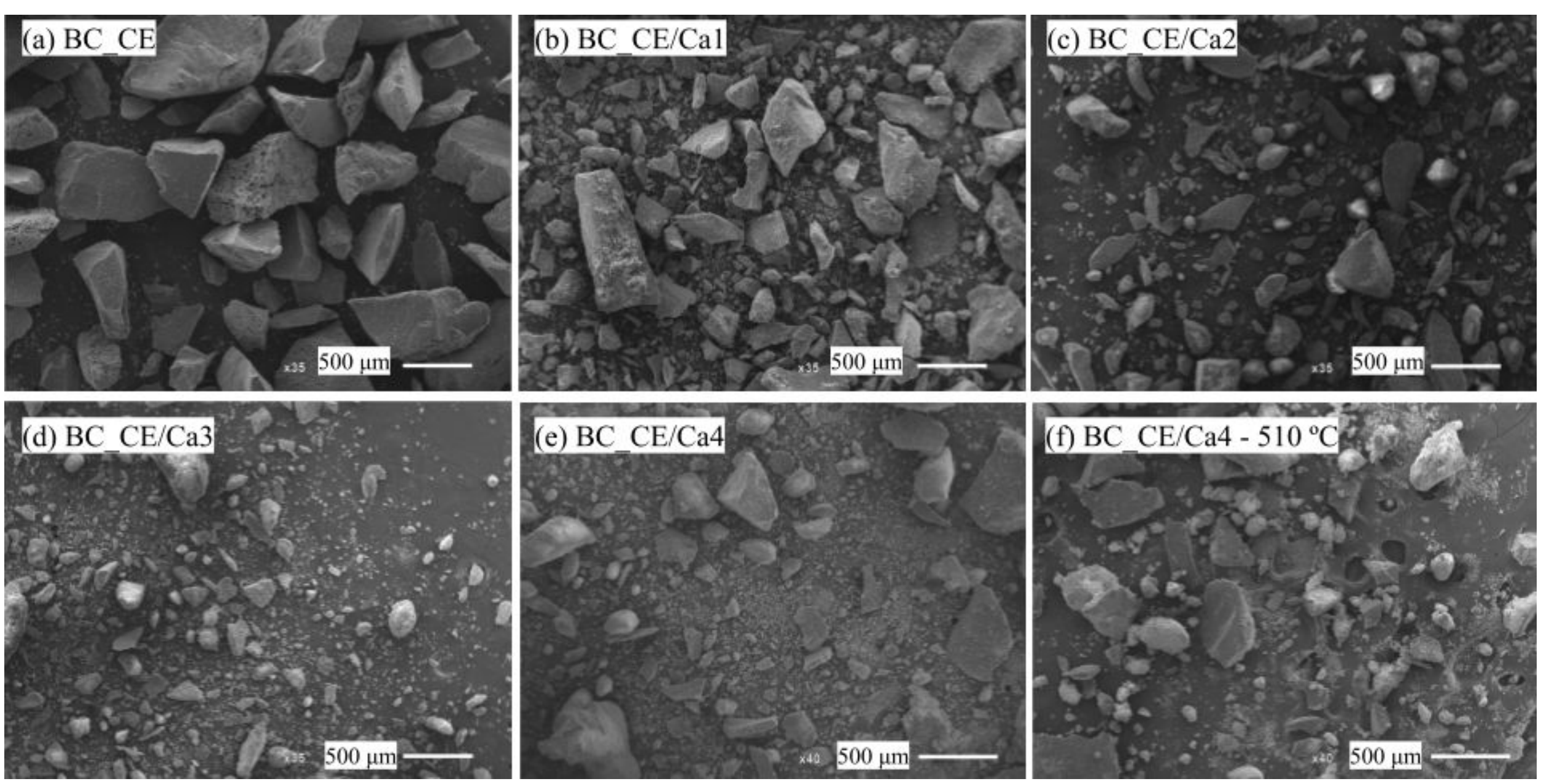

Figure S2. Results obtained from the SEM analysis for the samples of (a) BC_CE, (b) BC_CE/$Ca_1$, (c) BC_CE/$Ca_2$, (d) BC_CE/$Ca_3$, (e) BC_CE/$Ca_4$ and (f) BC_CE/$Ca_4$ after heat treatment at 510 ºC, at a magnification of 100 and 250×.

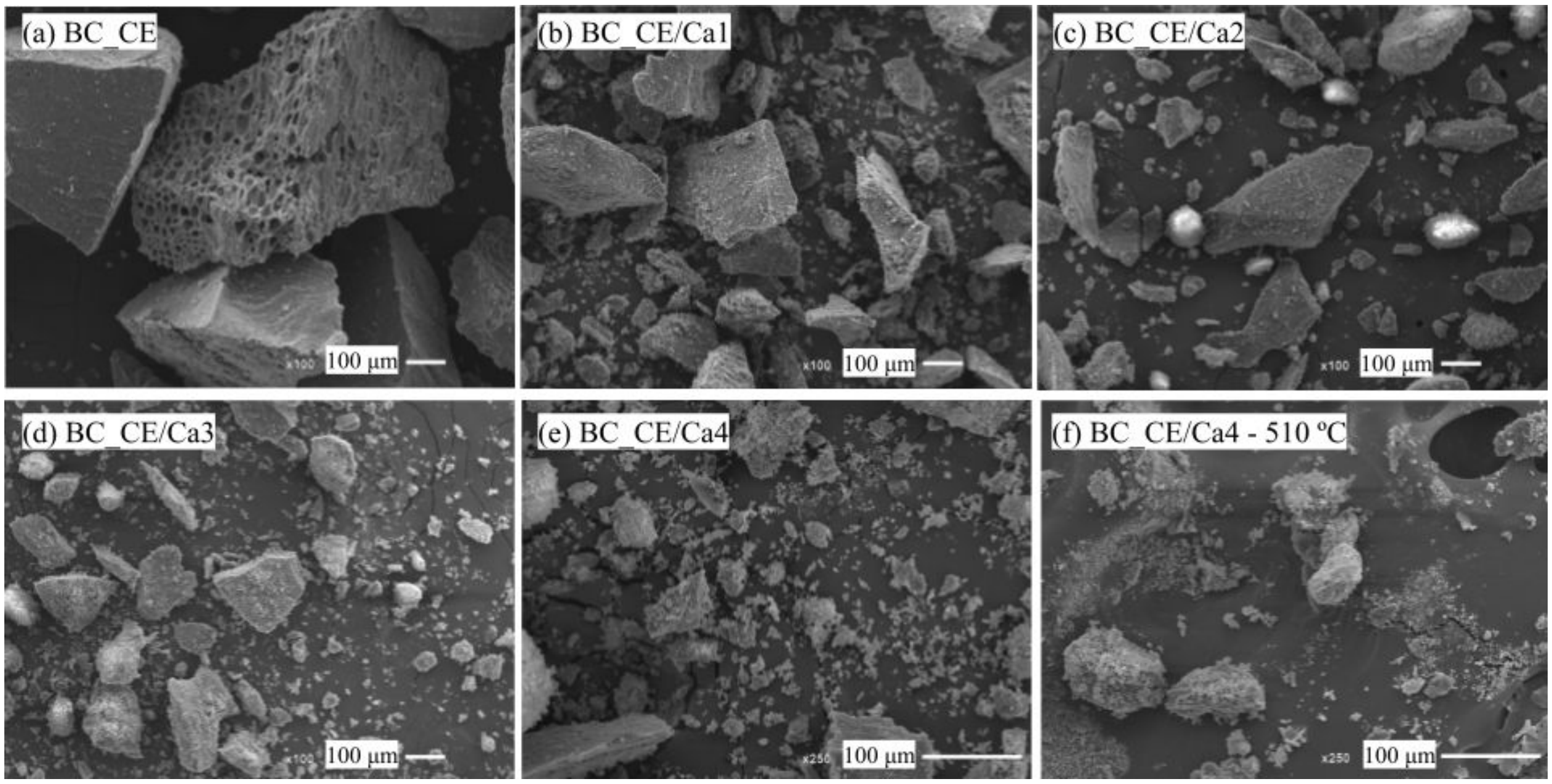


Figure S3. EDS elemental maps of the BC_CE sample, corresponding to the C and O elements, at 500× magnification.

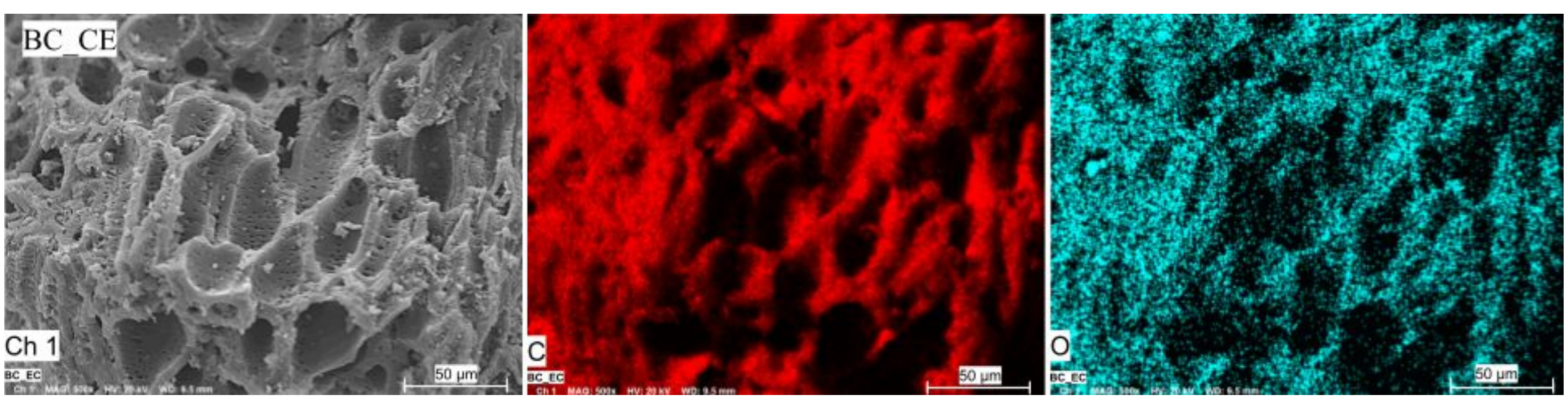

Figure S4. EDS elemental maps of the $BC\_CE/Ca_1$ sample, corresponding to the elements C, O, and Ca, at 500× magnification.

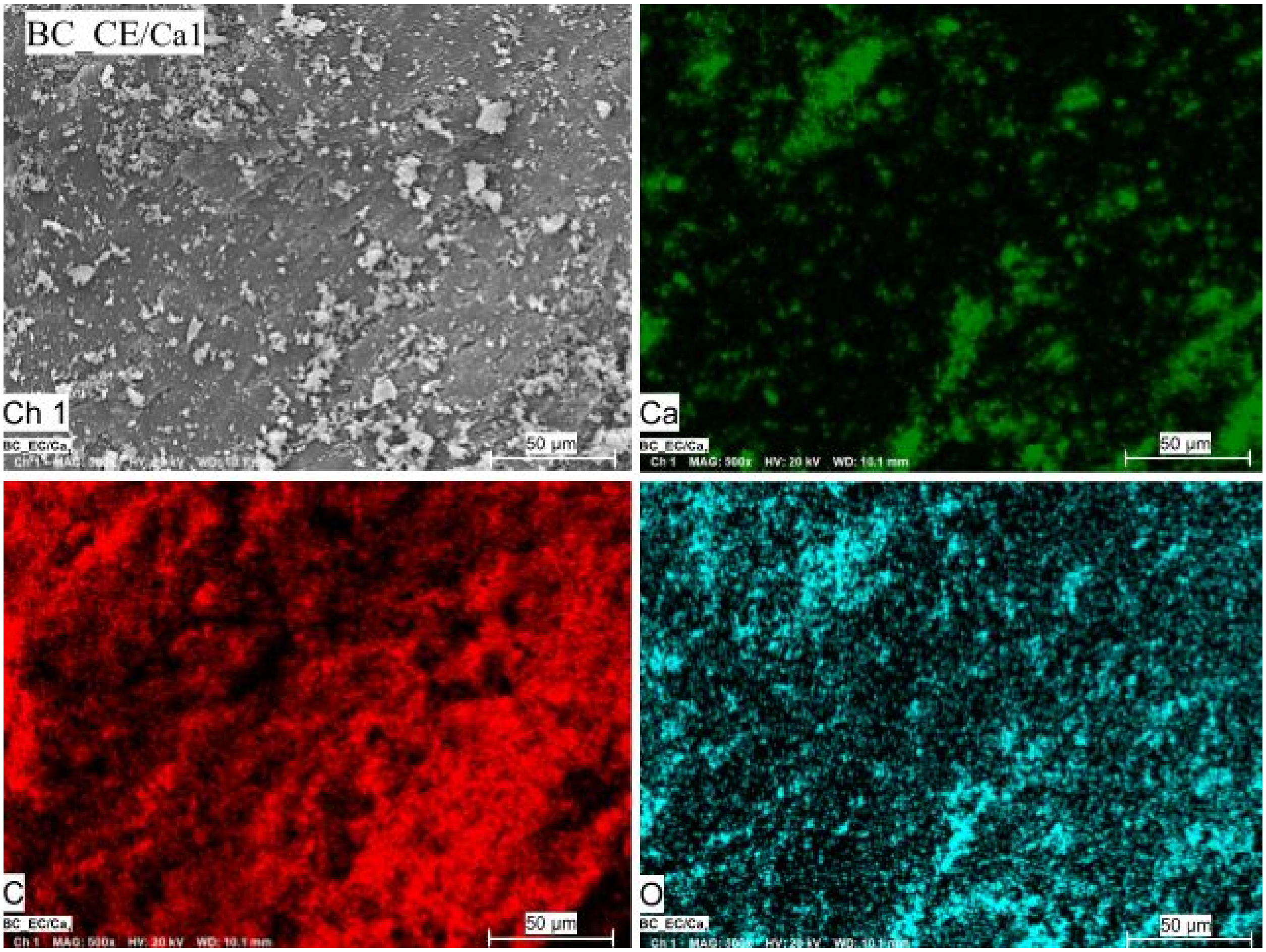

Figure S5. EDS elemental maps of the BC_CE/$Ca_2$ sample, corresponding to the elements C, O, and Ca, at 500× magnification.

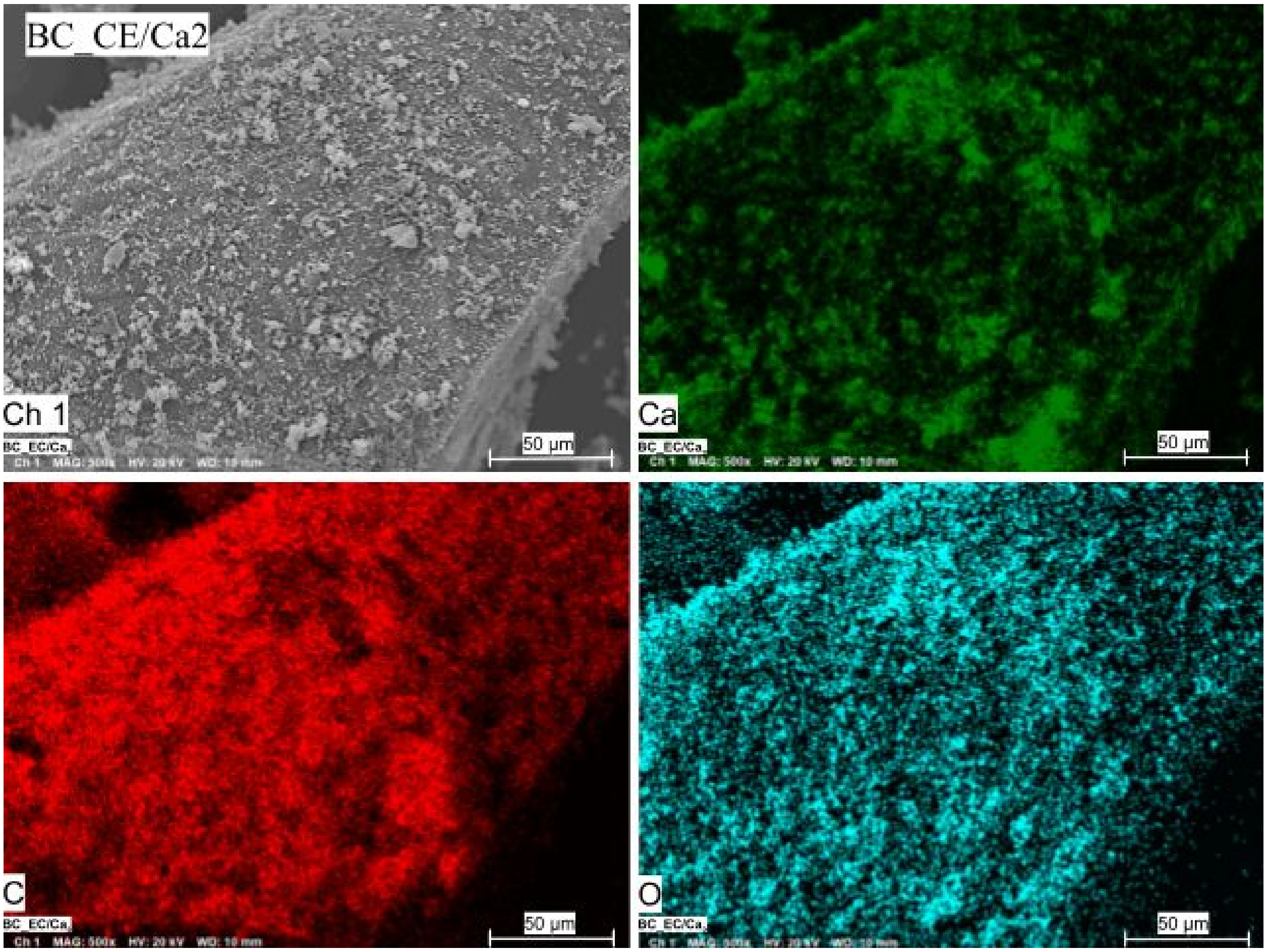

Figure S6. EDS elemental maps of the BC_CE/$Ca_3$ sample, corresponding to the elements C, O, and Ca, at 500× magnification.

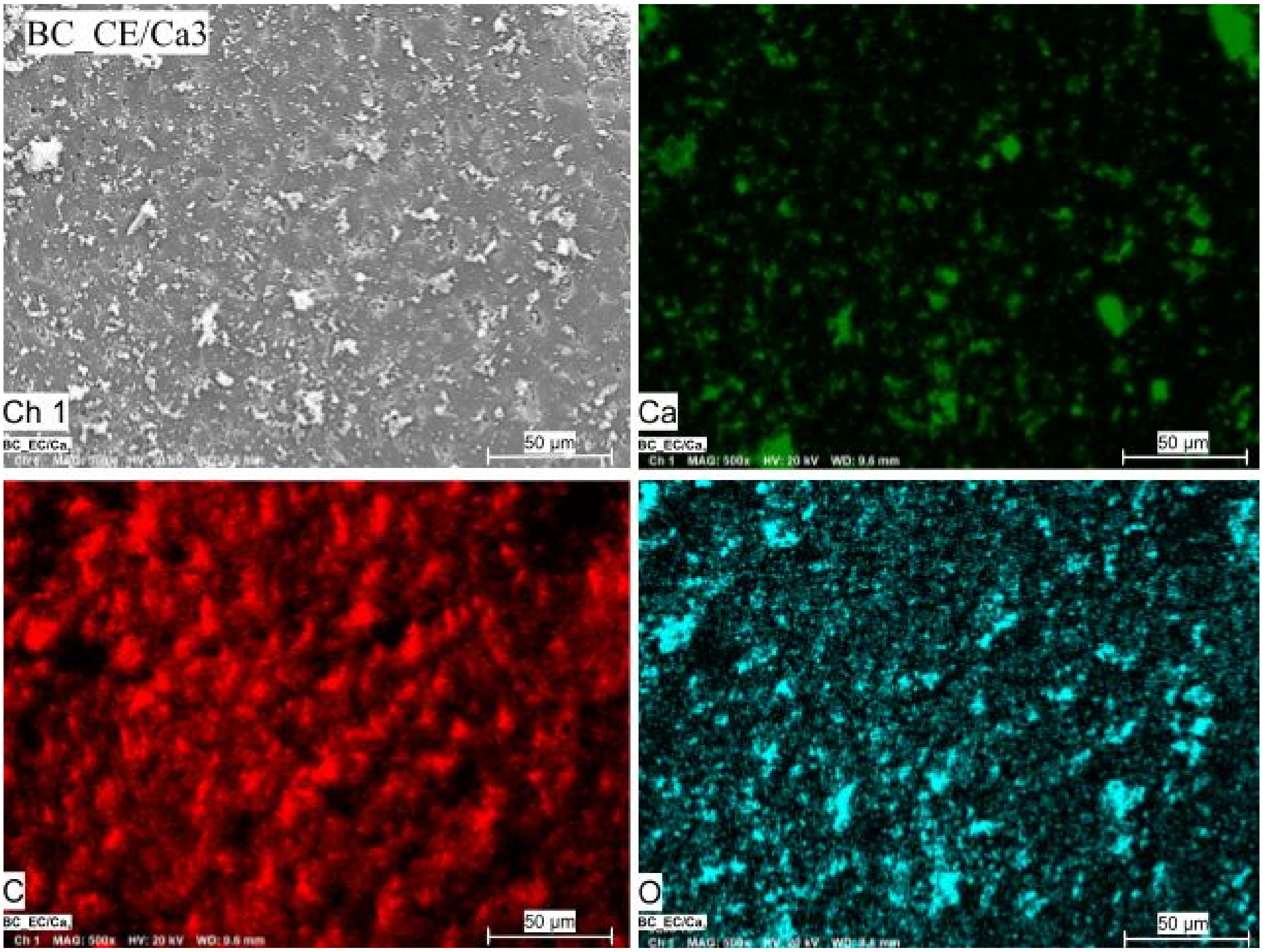

Figure S7. Comparative plot showing the SSA and $V_P$ values together with the Ca contents of the BC samples.

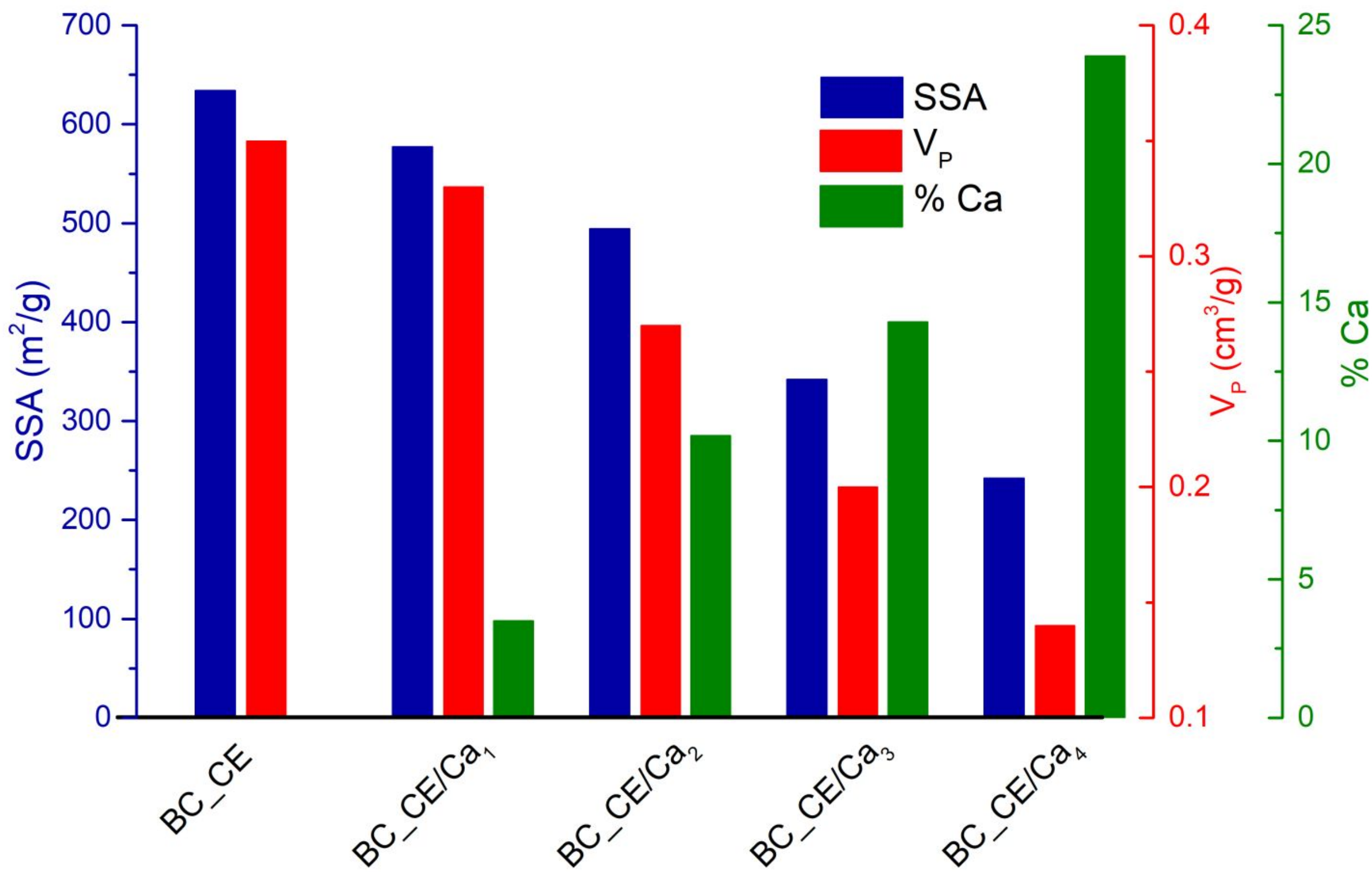

Figure S8. (a) $N_2$ adsorption/desorption isotherms and (b) pore size distribution of the BC_CE and BC_CE/$Ca_4$ samples.

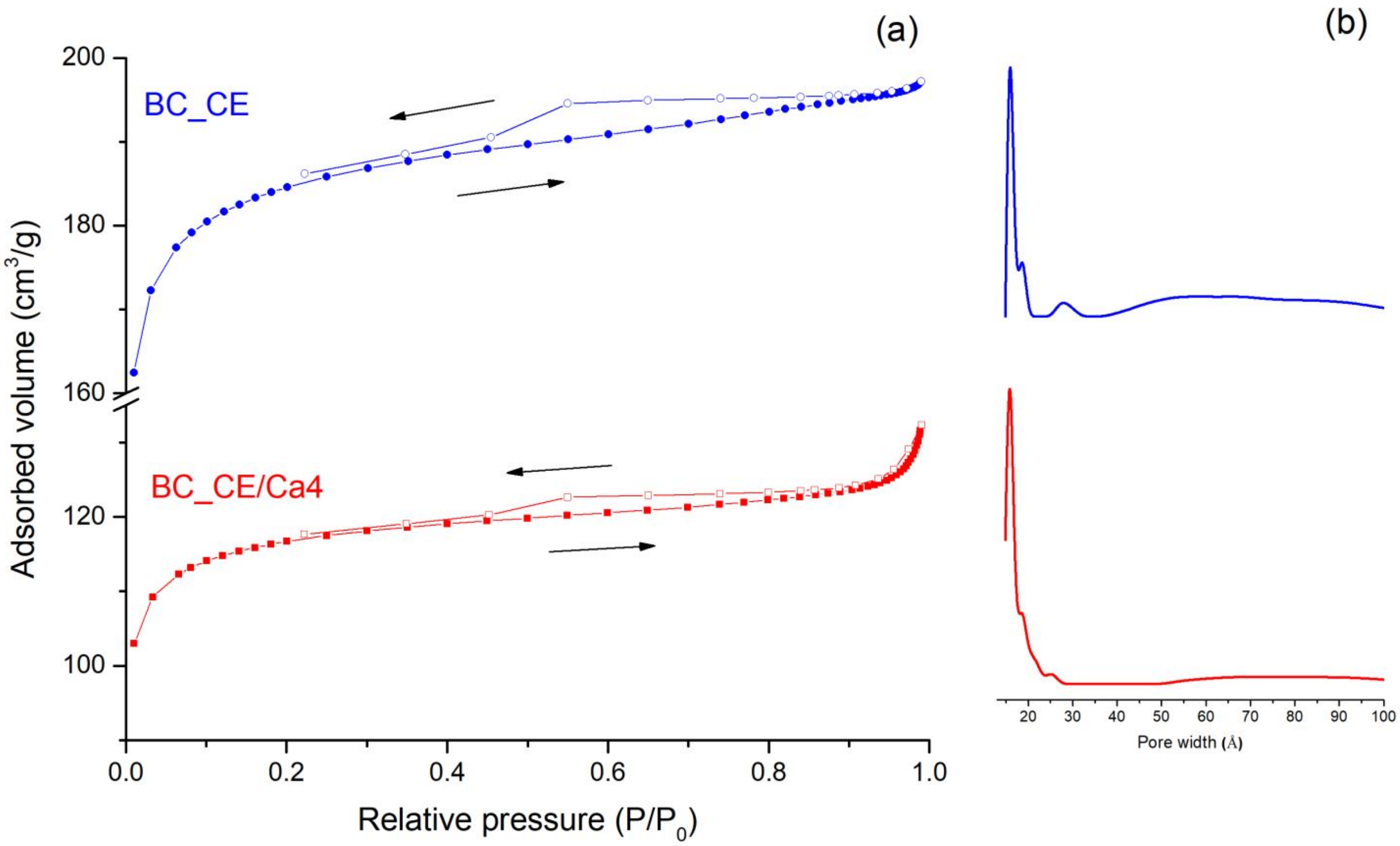

Figure S9. TG curves recorded during three consecutive cycles of $CO_2$ adsorption / desorption at 50 °C using the BC_CE and BC_CE/$Ca_4$ samples.

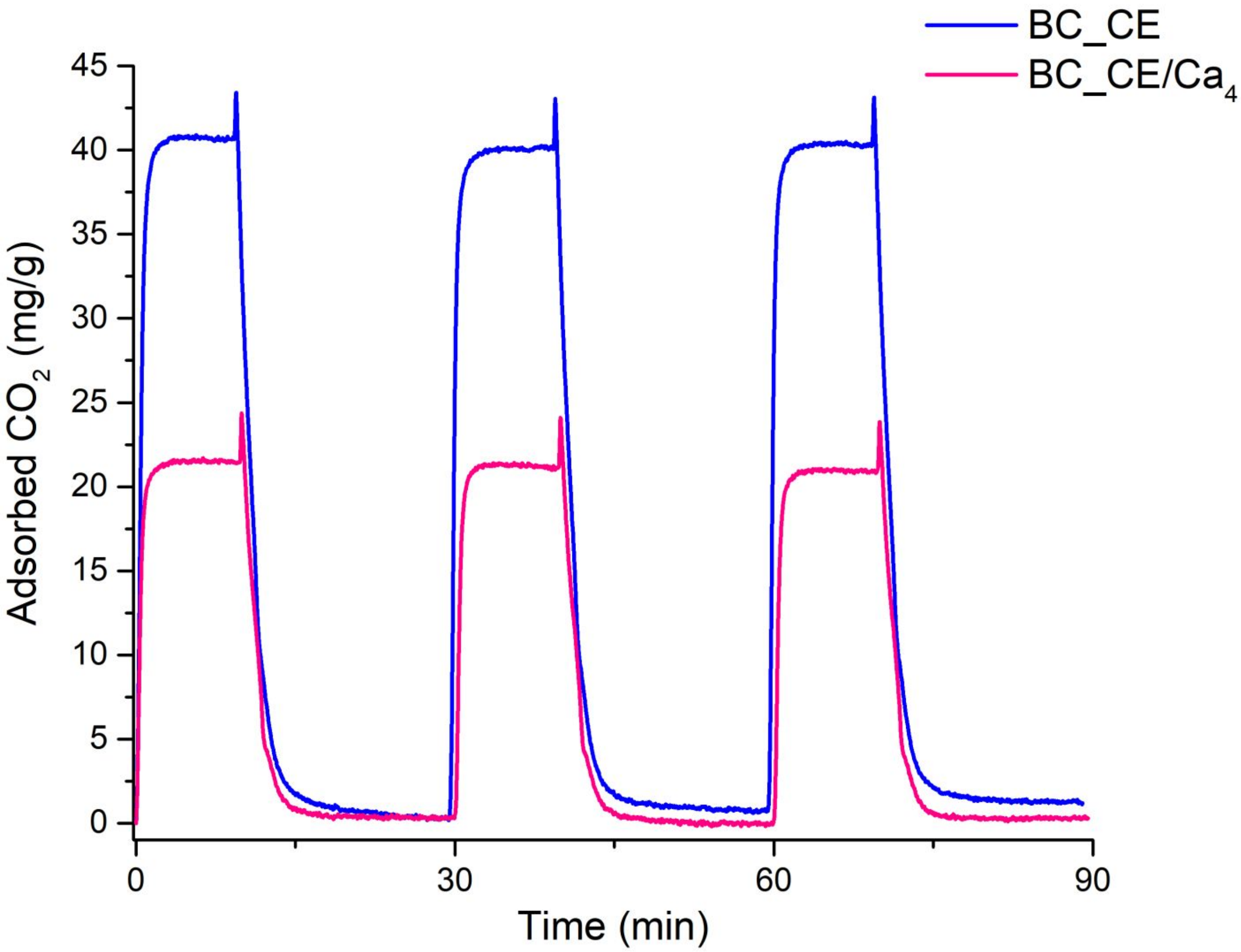

Figure S10. Fitting curves obtained using the pseudo-first order, pseudo-second order, and Avrami kinetic models for the $CO_2$ adsorption process on the BC_CE sample at temperatures of (a) 30, (b) 50, (c) 80, (d) 100, and (e) 120 ºC.

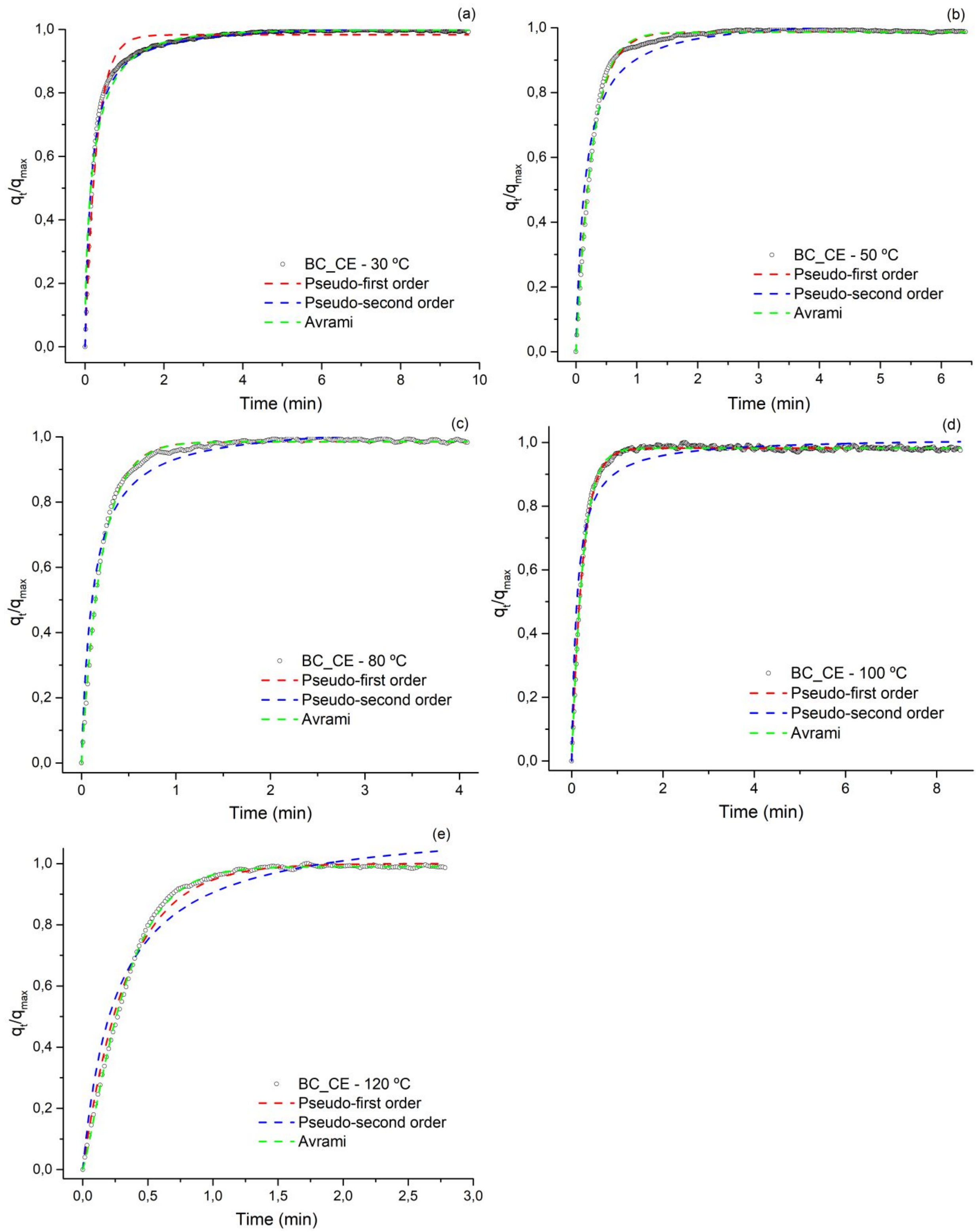

Figure S11. Fitting curves obtained using the pseudo-first order, pseudo-second order, and Avrami kinetic models for the $CO_2$ adsorption process on the BC_CE/$Ca_1$ sample at temperatures of (a) 30, (b) 50, (c) 80, (d) 100, and (e) 120 ºC.

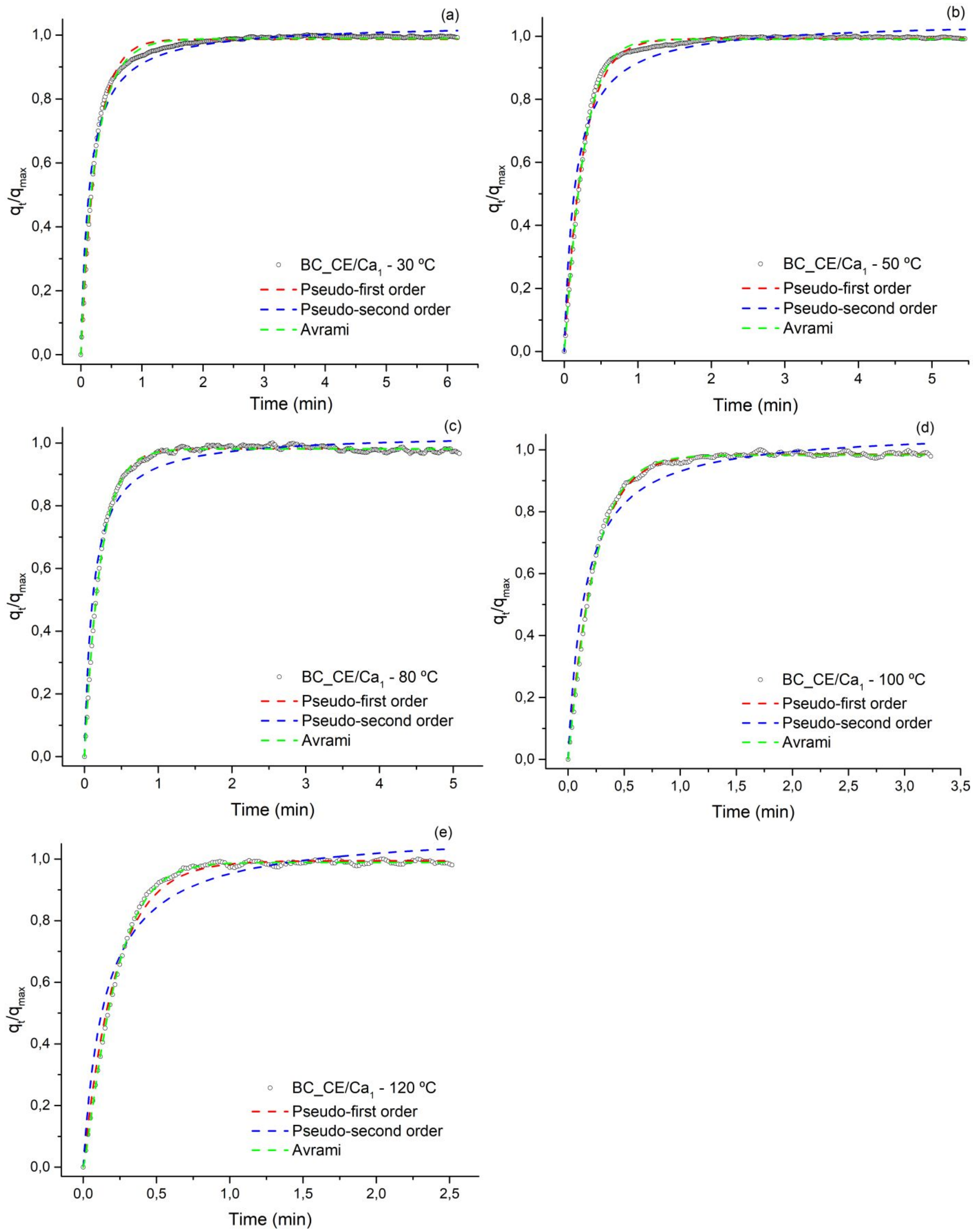

Figure S12. Fitting curves obtained using the pseudo-first order, pseudo-second order, and Avrami kinetic models for the $CO_2$ adsorption process on the BC_CE/$Ca_2$ sample at temperatures of (a) 30, (b) 50, (c) 80, (d) 100, and (e) 120 ºC.

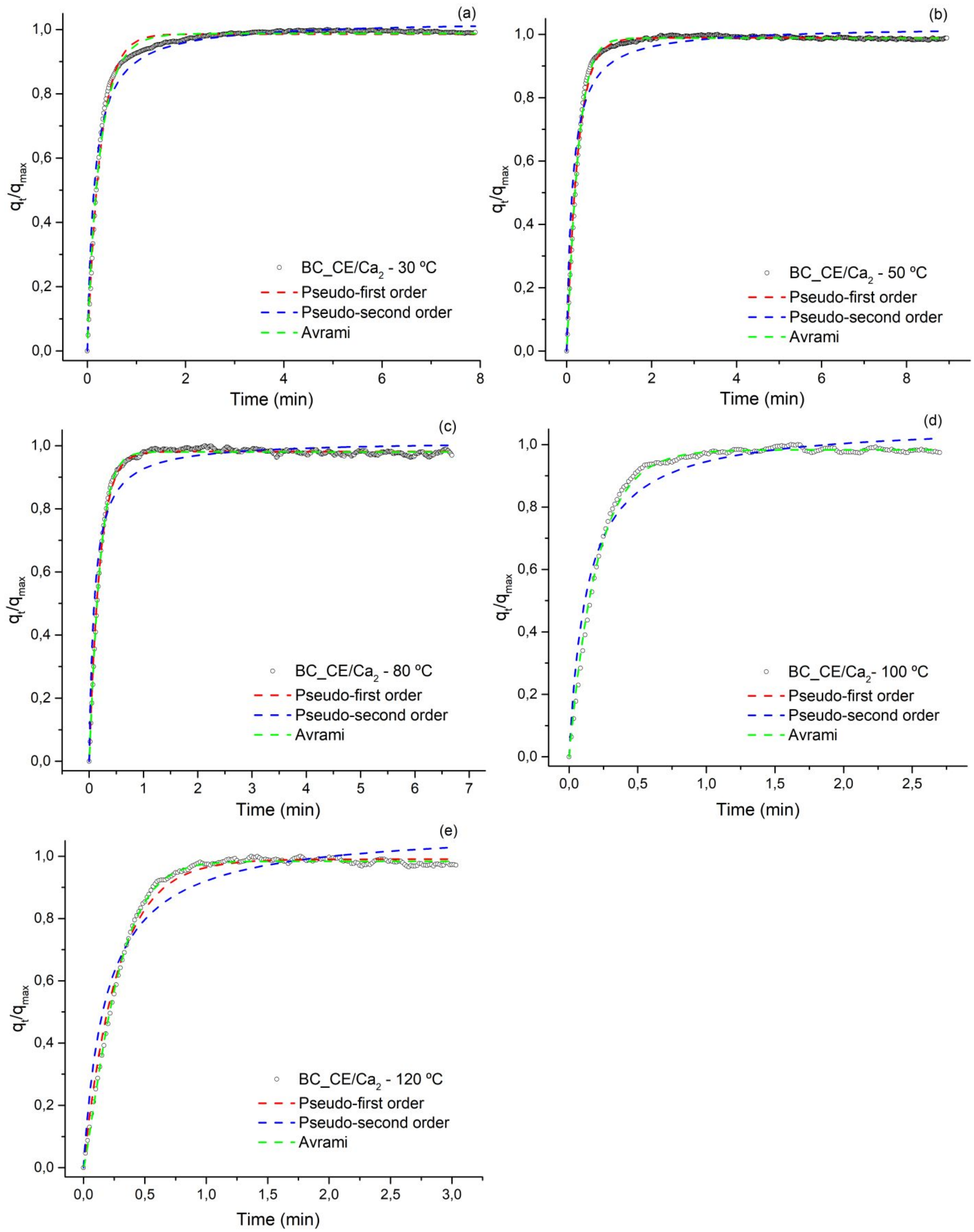

Figure S13. Fitting curves obtained using the pseudo-first order, pseudo-second order, and Avrami kinetic models for the $CO_2$ adsorption process on the BC_CE/$Ca_3$ sample at temperatures of (a) 30, (b) 50, (c) 80, (d) 100, and (e) 120 ºC.

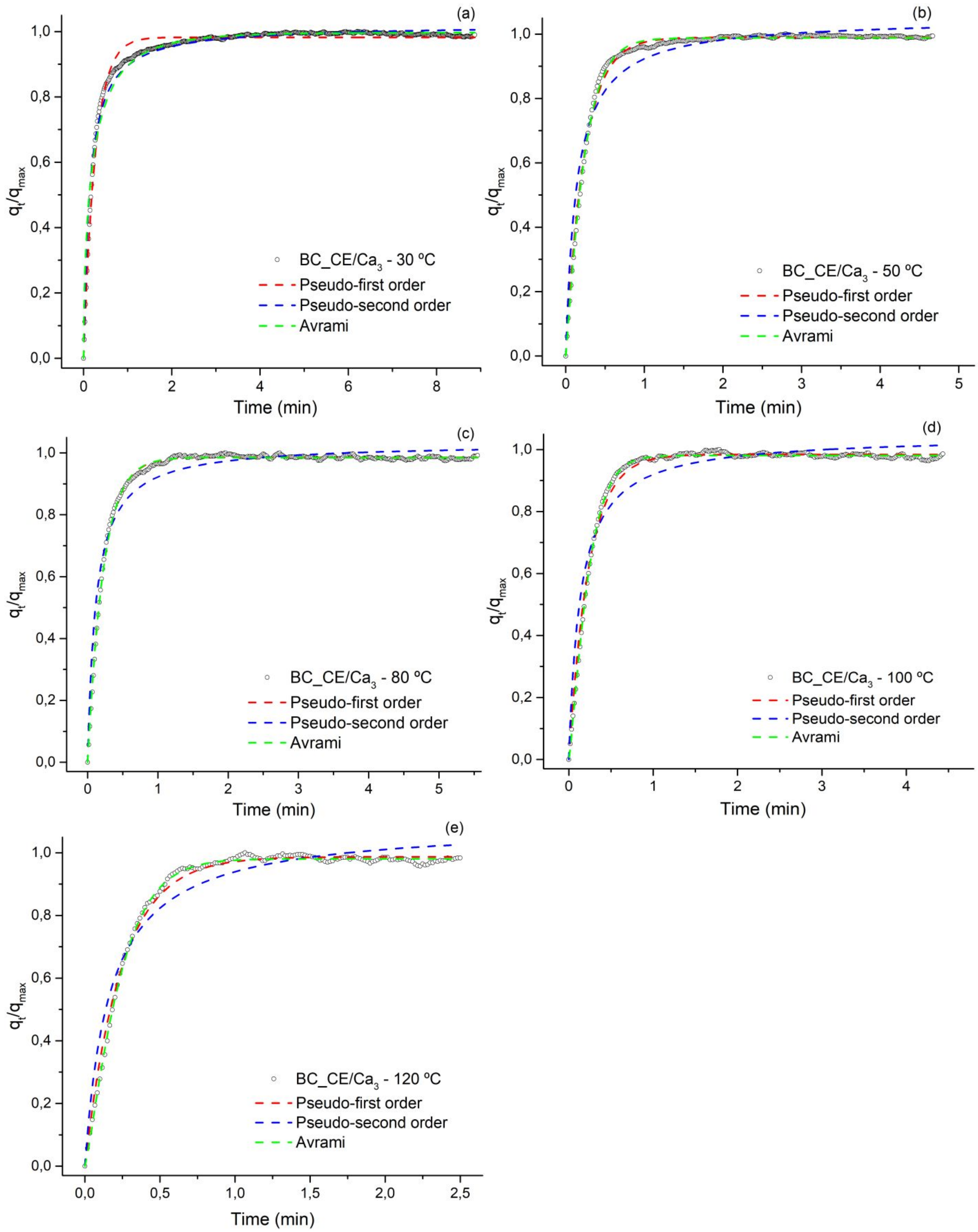

Figure S14. Fitting curves obtained using the pseudo-first order, pseudo-second order, and Avrami kinetic models for the $CO_2$ adsorption process on the BC_CE/$Ca_4$ sample at temperatures of (a) 30, (b) 50, (c) 80, (d) 100, and (e) 120 ºC.

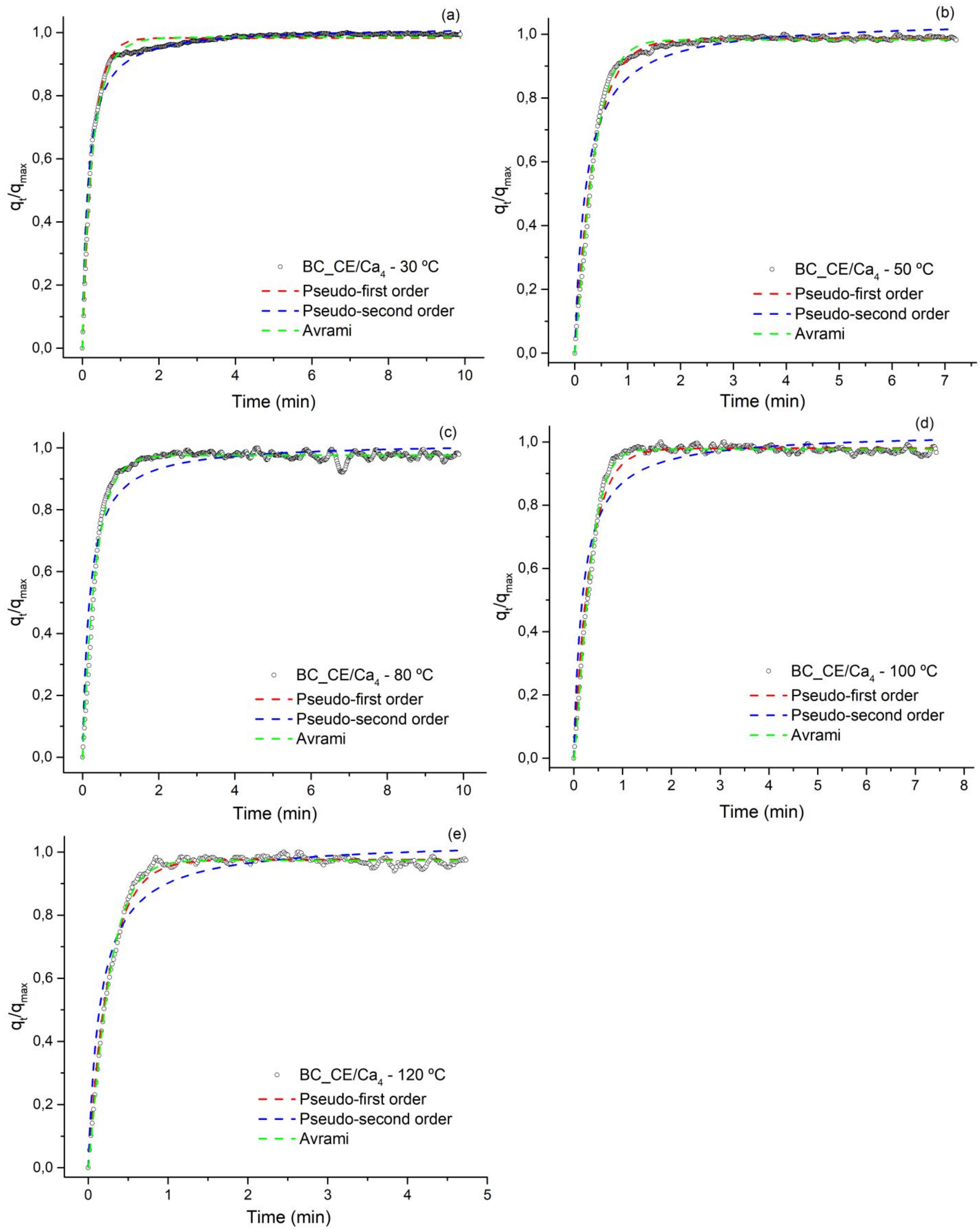

Figure S15. Avrami kinetic constant as a function of temperature for the samples: (a) BC_CE, (b) BC_CE/$Ca_1$, (c) BC_CE/$Ca_2$, (d) BC_CE/$Ca_3$, and (e) BC_CE/$Ca_4$.

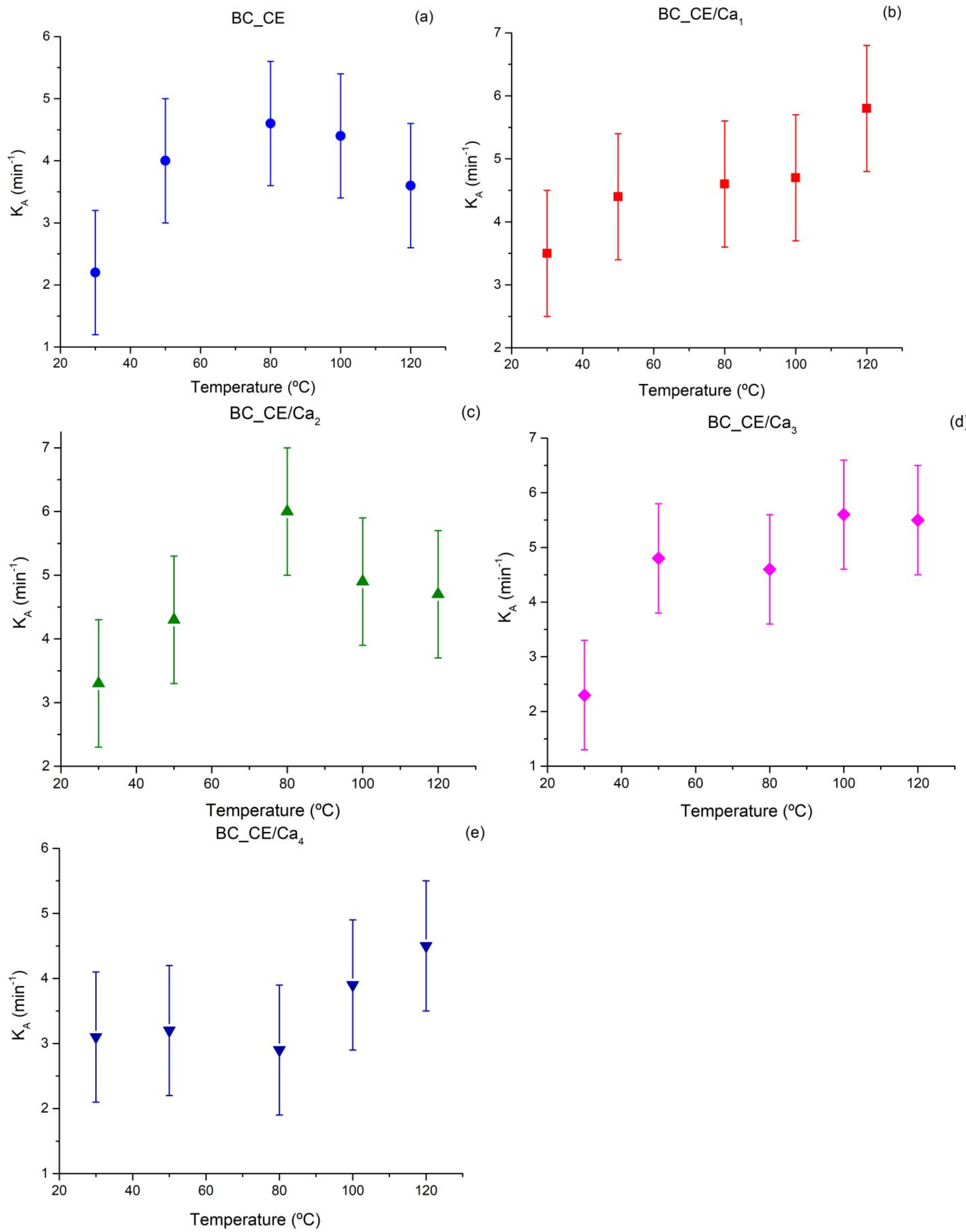

Figure S16. X-ray diffractograms obtained under conditions I (immediately after sample preparation), III (after $CO_2$ adsorption at 50 ºC), and IV (after the carbonation experiments at 510 ºC) for the samples: (a) $Ca(OH)_2$_synt, (b) BC_CE, (c) BC_CE/$Ca_1$, (d) BC_CE/$Ca_2$, (e) BC_CE/$Ca_3$ and (f) BC_CE/$Ca_4$.

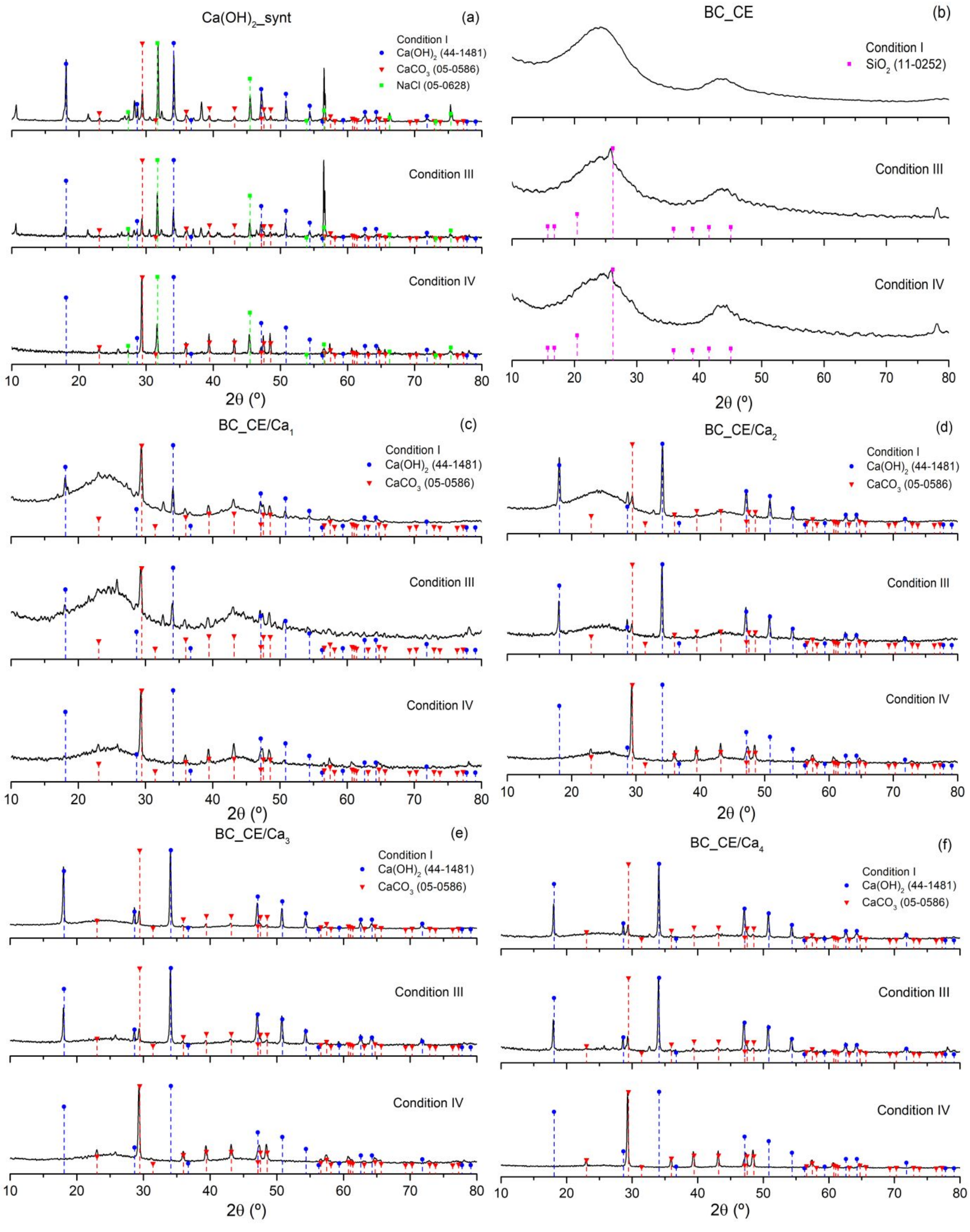

Figure S17. TG curve recorded for the BC_CE/$Ca_4$ sample after the carbonation experiment in a humid environment.

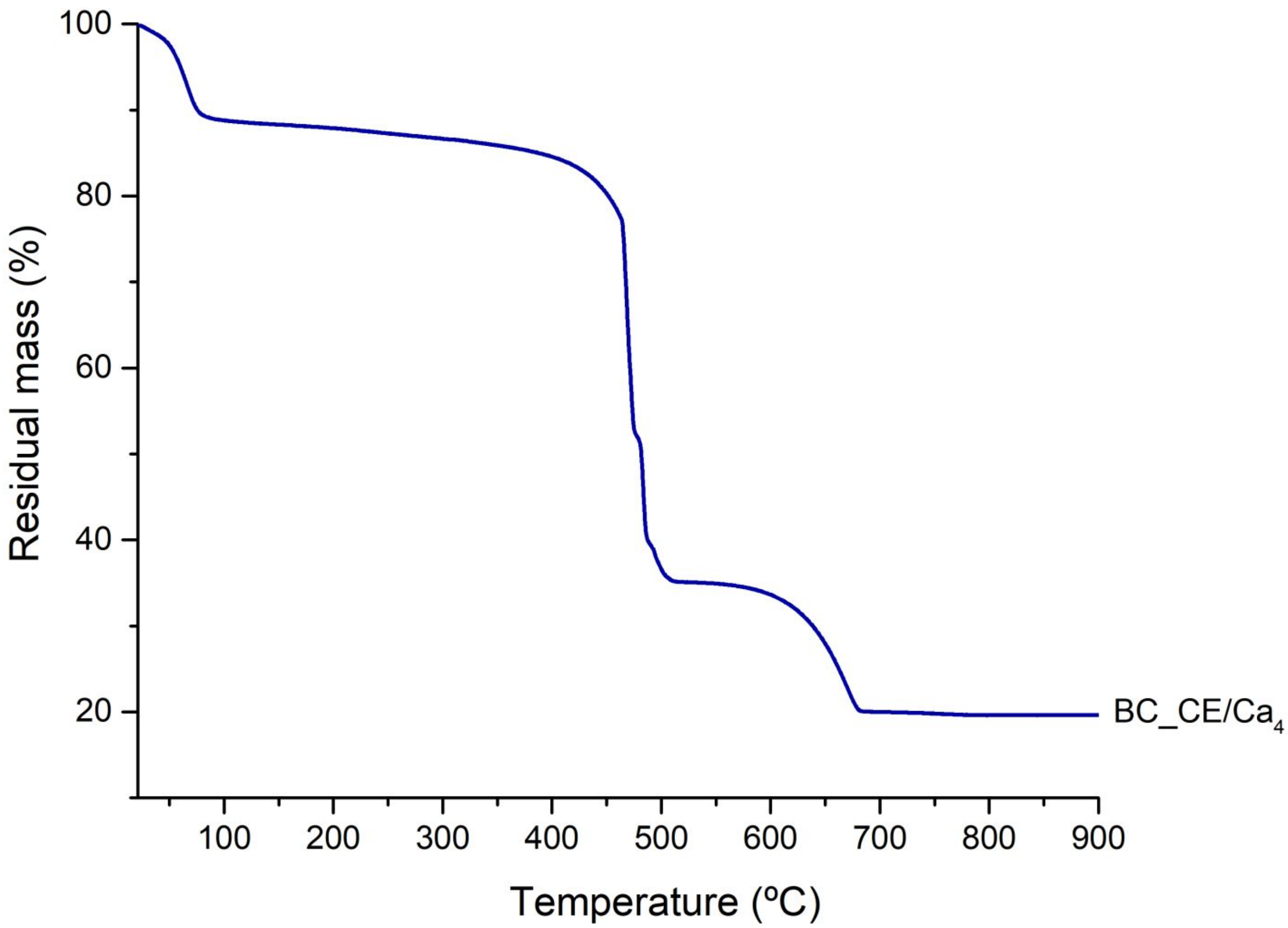

Supplement: Supplementary file 1 [file ao6c02339_si_001.pdf]
